# Monthly spatial dynamics of the Bay of Biscay hake-sole-Norway lobster fishery: an ISIS-Fish database

**DOI:** 10.1038/s41597-022-01408-0

**Published:** 2022-06-15

**Authors:** Audric Vigier, Michel Bertignac, Stéphanie Mahévas

**Affiliations:** 1 DECOD (Ecosystem Dynamics and Sustainability), IFREMER, INRAE, Institut Agro - Agrocampus Ouest, Nantes, France; 2 Queen’s University Belfast, School of Biological Sciences, Belfast, United Kingdom; 3 UK Centre for Ecology & Hydrology, Penicuik, United Kingdom; 4 DECOD (Ecosystem Dynamics and Sustainability), IFREMER, INRAE, Institut Agro - Agrocampus Ouest, Brest, France

**Keywords:** Ecological modelling, Population dynamics, Environmental sciences

## Abstract

We propose a database to describe the Bay of Biscay mixed demersal European fishery over the period 2010–2020 for the ISIS-Fish simulation tool. It was built upon national and European fishing databases, scientific survey datasets, models estimates, literature, and the formulation of assumptions. It accounts explicitly for spatial and seasonal processes, and for mixed fisheries phenomenons. This database encompasses population dynamics for 3 stocks, hake, sole and Norway lobster, exploitation dynamics for numerous fleets and *métiers*, and the description of current fishing management, as well as fishers adaptation. A calibration procedure was designed to ensure consistency between all these diverse and heterogeneous parameters compiled and estimated in the ISIS-Fish database and to ensure the model reproduces closely historical catch patterns. This database is a starting point towards operational simulations, of use for understanding the functioning of the fishery, the assessment of management strategies, or delivering short and long-term scenarios. It can be used to reproduce historical catch patterns, with room for improvement on some inter-annual and spatial dynamics.

## Abstract (summary)

**Table:** 

Measurement(s)	Fisheries simulation database
Technology Type(s)	Data collection and curating, modelling
Sample Characteristic - Organism	Merluccius merluccius • Solea solea • Nephrops norvegicus
Sample Characteristic - Environment	Mixed demersal fisheries
Sample Characteristic - Location	Bay of Biscay

## Background & Summary

The Bay of Biscay is a very productive area, home to a set of widely complex, mixed fisheries, with more than 200 commercial species. Among them is the Bay of Biscay demersal mixed fishery, involving numerous populations, and interactions between fishing fleets and populations complicating its management^1^.

This European fishery is currently managed to reach Maximum Sustainable Yield (MSY) objectives, under the European Union Common Fisheries Policy^2^, involving single-stock management measures, such as catch or landings quotas (Total Allowable Catch or Landings, respectively TACs and TALs), Minimum Conservation Reference Sizes (MCRS), technical measures, such as minimal mesh sizes, and the progressive setup, since 2015, of the Landings Obligation (LO), aiming at reducing discards.

This single stock approach to management however ignores mixed aspects of the fishery, such as technical interactions, *i.e*. interactions between several stocks through various fishing activities targeting or by-catching simultaneously these stocks^3^. Hence, the management targeted at the exploitation of one of these stocks may impact the other stocks through change in fishing activities. Another key factor in the assessment of stocks and fishing pressure is their spatial distributions’ heterogeneity, despite being poorly accounted for. Also, the spatial effects of management measures remain poorly understood, requiring a more thorough investigation.

We focused on 3 stocks of the Bay of Biscay mixed demersal fishery: northern hake (*Merluccius merluccius*) - sole (*Solea solea*) - Norway lobster (*Nephrops norvegicus*) in ICES divisions 8a, 8b and 8d. They were the most caught stocks in value in 2015 in this area^1^ and interact in a highly mixed demersal fishery. They are at the core of ecosystem based management approach within the context of MSY, good environmental status as defined by Marine Strategy Framework Directive^4^ (MSFD) and biodiversity conservation. Various fleets and *métiers* (here meant as a combination of a gear, a location and mix of target species) may catch these species, as target or bycatch, with possibilities of effort re-allocation between them. These 3 species are subject to single-stock TACs and TALs, MCRS, constraints on mesh sizes, and since 2016 Landings Obligation (LO; hake and sole only). Technical interactions emerge between populations, the strongest being due to Norway lobster trawlers in the Great Mudbank, a hake nursery, causing high discards of undersized hakes^5^. Such interactions increase choke species risk under LO^6^.

An operational simulation model is needed to overcome these issues. We propose a database, which is a necessary step towards the simulation of the fishery’s dynamics, accounting explicitly for spatial features and technical interactions, developed under the generic fisheries simulation model ISIS-Fish^7,8^, applied in a wide variety of fisheries globally^9–16^ to assess the efficiency of fishing policies to reach the UN Agenda for Sustainable Development 2030 objectives^17^. In ISIS-Fish, a regional database fully describes the fisheries dynamics in 3 modules - populations, exploitation and management - with flexibility, to adapt to available knowledge. They are spatially explicit and modelled with a monthly time step.

Building an ISIS-Fish database consists in informing the simulation tool with the values of each fishery dynamics parameter, with a possibility to add custom assumptions, and to calibrate the model to a set of observations. Therefore, it is necessary to collect parameters values of population dynamics for each marine species (stock), of fishing activities dynamics for each fleet, and of management dynamics for each established policy of the studied fishery.

This paper describes the ISIS-Fish regional database of the hake-sole-Norway lobster fishery in the Bay of Biscay. This database is a synthesis of data and knowledge gathered for simulation purposes to investigate the functioning of the fishery and management decision-making, of use for modellers community within research projects (e.g. MACCO^18^, SEAWISE^19^) and management bodies.

**Fig. 1:**
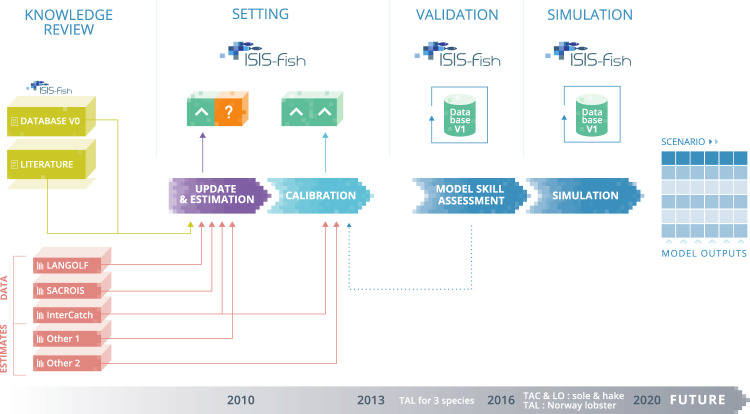
Summary workflow leading to the updated ISIS-Fish database. This figure encompasses knowledge review, parameter settings, validation and simulation steps. An updated database was set up from knowledge review (literature and use of a former database in mustard boxes, datasets in salmon boxes), as illustrated by the purple arrow. Among its parameters, some values were known (green box) or unknown (orange box). The values of the latter were set through calibration (light blue arrow), using available datasets. The simulation model went through a skill assessment, which required to loop back to assumptions writing and re-calibrating the model (dotted arrow), prior to performing a simulation (blue arrows). The simulation time series (grey arrow) displays the calibration period (2010–2012) and the simulation period (2013–2020), as well as periods of quotas (TAL for landings, TAC for catch) and landings obligation (LO) implementation (each year marking the 1^*st*^ January).

This paper describes, firstly, the set of parameters and assumptions included in the regional database of the mixed demersal fishery of the Bay of Biscay, secondly, the reference simulation of the dynamics using ISIS-Fish and this regional database, and thirdly, the validation of the database and base simulation ability to reproduce the historical dynamics over the time period 2010–2020. Parameters were collected by reviewing available knowledge in scientific and grey literature, and by estimating missing values with available observations on the fishery as shown in Fig. 1.

## Methods

We took as a starting point the hake - sole - Norway lobster Bay of Biscay ISIS-Fish database used for COSELMAR project^16,20^ (see http://isis-fish.org/download.html section “Bay of Biscay scenario dataset”, Database V0 in Fig. 1). This database was built using 2010 data, and was not calibrated, as it was designed for a geo-foresight study. Since our aim was to describe the system over a decade and simulate realistic dynamics close to available observations to assess management measures, we needed to update the parametrisation and calibrate the database. We took 2010–2012 as the calibration period, and 2013–2020 as the simulation period (grey arrow Fig. 1). The database has a monthly temporal resolution (constrained by the ISIS-Fish framework) and the spatial scale was set to match ICES statistical rectangles (0.5° latitude by 1° longitude rectangles, defined by the International Council for the Exploration of the Sea (ICES) https://www.ices.dk/data/maps/Pages/ICES-statistical-rectangles.aspx), consistent with available knowledge and data.

In this section, we firstly describe all the data sources used to update and calibrate the database. Then, for each main component of an ISIS-Fish database - *i.e*. populations, exploitation and management - we describe this paper’s database parameters and assumptions. We finally describe the calibration procedure (inspired by previous work^21,22^), of which some results are shown in the Technical Validation section. We summarized this workflow in Fig. 1.

### Data sources

Data sources, estimates, and literature (including grey literature) were needed to update and calibrate the model. They are marked in Fig. 1 with salmon (data sources and estimates) and mustard (literature) blocks:

- SACROIS^23^: French landings and effort logbook declarations for 2010 were made available at the log-event*commercial category*ICES statistical rectangle*population scale. It was used to design exploitation features of the database, as well as populations spatial structure.
- LANGOLF survey: 2006–2010 LANGOLF surveys observations for 2006–2010 were made available for Norway lobster. They were used to work on Norway lobster abundance per length class and sex.
- Intercatch: catch observations for 2010–2020 in the Bay of Biscay for hake, at the quarter-*métier* group scale, and catch observations per class for sole on 2010–2012, and 2010 Norway lobster catch observations per sex and length class^24^, used to describe the inter-annual effort dynamics, to calibrate and validate the model.
- Estimates of hake abundance per size class in 2010, and hake quarterly estimates of recruitment on 2010–2012 from a northern hake spatial stock assessment model^21^, used to inform hake biology assumptions (named Other 1 in Fig. 1).
- ICES WGBIE^24^ 2010 estimates of abundance per class (sole and Norway lobster), to inform their abundance at the initial time step; 2010–2012 yearly fishing mortality estimates per age class (sole) to calibrate the database (named Other 2 in Fig. 1).
- Other population, exploitation and management assumptions were informed with scientific literature^25^ and grey literature^26,27^ (Literature block in Fig. 1).
- Management assumptions were informed with legal texts^2,4,28–34^ and reported quota values in working group reports^24^.

### About populations

This section describes for each species the assumptions and parameters values, except for accessibility, which has been calibrated, as described in section Calibration procedure. For all assumptions and values, more details are provided in Supplementary Information’s section 2.2.

#### Hake

The stock size structure was defined with 1 cm size bins for [1;40[cm individuals, 2 cm for [40;100[cm individuals, and 10 cm for [100;130+] cm individuals^35^. Areas of presence were defined based on 2010 SACROIS French landings data per commercial category and statistical rectangle^23^, leading to the definition of a presence, a recruitment, an interim recruitment and a spawning area^25^ (see Supplementary Information’s section 2.2 and Figure S1). These areas allow for the description of intra-Bay of Biscay migrations related to spawning and recruitment processes: mature individuals aggregate at the beginning of the year on the shelf break to spawn, and then disperse on the shelf^36–40^ (at the beginning of April and July in the model). Also, from age 1 (around 20 cm), individuals in recruitment zone spread in interim recruitment zone, to model a diffusion towards areas neighbouring the nursery area, at the beginning of each time step (see Supplementary Information’s section 2.2 and Table S11). Maturity-at-size and weight-at-length relationships were the same functions as used by ICES working group^35,41^. Natural mortality was fixed at 0.5, basing on preliminar runs, instead of the commonly used 0.4^42^. Recruitment values were defined prior to the simulation for 2010–2020 using available estimates on the 2010–2015 time series^21,27^. Deterministic estimates from these sources were allocated to the recruitment area in the Bay of Biscay and the beginning of each month in January-September on the whole time series, of which values are provided in the Supplementary Information’s section 2.2 and Table S3. Growth is modelled through monthly growth increments^5,25^. However, given the different widths of size bins in the implemented size structure, a correction was provided to values in the transition matrix to eliminate artifacts when growing to a size bin wider than the size bin of origin, as detailed in Supplementary Information’s section 2.2. Abundance at the initial step in each zone was estimated from Bay of Biscay abundance estimates for 2010^21^. Mature individuals over 20 cm were allocated to the spawning area, all individuals strictly shorter than 20 cm were allocated to the recruitment area (as they were assumed to be less than 1 year old), and remaining individuals were allocated to the interim recruitment area. None were allocated to the presence area, in which individuals will go later in the time series, after disaggregating from the spawning area^25^ (Table S13).

#### Sole

The stock is age structured, with 7 classes going from ages 2 to 7+^43^ (Table S2). No seasonal variations were implemented. Only a single presence zone was defined (see Supplementary Information’s section 2.2 and Figure S1), as in preliminary runs defining more presence areas for sole did not yield more knowledge in this study. We implemented ICES working group values for natural mortality, weight-at-age (Table S1) and maturity-at-age^43^. Recruitment occurs at the beginning of each year, individuals being recruited at age 2 (ages 0–1 were not modelled; Table S4). We implemented ICES working group estimates^27^ for abundance at initial time step (Table S14).

#### Norway lobster

The stock has a sex-size structure, with 1 mixed recruitment class at 0 cm; 33 length classes for males at 2 carapace length mm intervals, from [10;12[to [72;74[carapace length mm; 23 length classes for females at 2 carapace length mm intervals, from [10;12[to [52;54[carapace length mm. A single presence area was defined: the Great Mudbank^21^ (see Supplementary Information’s section 2.2 and Figure S1). Several seasonal processes occur for this stock, impacting recruitment, accessibility and growth: 1/ January, begins with the annual recruitment. Females are inside their burrows, less accessible; 2/ February-March females are inside their burrows, less accessible; 3/ April: Spring moulting, females are more accessible; 4–5/ May-August females are more accessible; 6/ September, females are inside their burrows, less accessible; 7/ October: Autumn moulting only for immature females and all males, females are inside their burrows, less accessible; 8/ November-December, females are inside their burrows, less accessible^44^. We implemented ICES working group values for natural mortality, weight-at-class and maturity-at-class^45–47^. Growth occurs twice a year, when moulting in April and October, and is modelled with growth increments. Recruitment occurs at the beginning of each year, modelled with a Beverton-Holt relationship^26^, and was assumed to have the same spatial distribution as spawning stock biomass. Abundance at initial step was derived from LANGOLF survey observations and ICES WGBIE estimates^25,26^ (Table S16).

### About exploitation

The fishing exploitation structure (fleets, strategies, *métiers* and gears) were derived following a classification method on SACROIS 2010 landings and effort data^13,23^ from French fleets, and taken from a TECTAC project (https://cordis.europa.eu/project/id/Q5RS-2002-01291) database for Spanish trawlers. More details on their definition are provided in Supplementary Information’s section 2.3, Tables S5–S9 and S20–S21 and Figure S3. Spanish longliners and gillnetters fleets exploitation was described based on catch (observations from Intercatch^48^) rather than effort.

Hake selectivity and discarding functions (one for each gear) were taken from estimates of a spatial hake stock assessment model^21^. Parameters values and formulæ are provided in Supplementary Information’s section 2.3 and Tables S6-S7. On top of this, inter-annual fleet dynamics factors were included in equation (21) of ISIS-Fish documentation^8^ in order to account for observed catch temporal variations. These factors are therefore multiplicative parameters of the target factor of each species for each *métier*. They are computed using observed catch^27^ and differ according to the period and targeted species:

- over 2010–2016, it is a ratio of observed catch in weight per year over catch observations for 2010: for hake, one per *métier* *season*year $\left(\frac{ObservedCatc{h}_{metier,season,year}}{ObservedCatc{h}_{metier,season,2010}}\right)$, for sole, one per *métier* *year $\left(\frac{ObservedCatc{h}_{metier,year}}{ObservedCatc{h}_{metier,2010}}\right)$, and for Norway lobster, one per year (identical for each *métier* catching Norway lobster) $\left(\frac{ObservedCatc{h}_{year}}{ObservedCatc{h}_{2010}}\right)$;
- over 2017–2020: at the time of writing these assumptions, more recent data was not available, and ratios were deduced from trends on 2014–2016. A linear model was fitted on ratios deduced earlier on 2014–2016. If a significant trend was identified (hake: whitefish trawlers quarters 2 and 4, longliners and gillnetters seasons 2–3; sole and Norway lobster: all *métiers*), the slope was used to deduce 2017–2020 ratios (the slope was halved for hake whitefish trawlers and sole and Norway lobster values to avoid unrealistic high values of effort). Otherwise, 2016 ratios were used.

All values are provided in Supplementary Information’s section A.2 Tables S22–S24, and the final values of target factors are derived from the Calibration procedure.

### About management

We implemented a set of management rules close to what is currently implemented in the Bay of Biscay.

All stocks are managed by TALs (Total Allowable Landings) until 2015 and then by TACs (Total Allowable Catch), except for Norway lobster, managed by TALs on the whole time series, not being under the landings obligation. To favour a better parametrisation, allowing for more reliable dynamics on the following years of the time series, no TALs were implemented during the calibration period (2010–2012; Fig. 1). These regulations were implemented from 2013 using historically TALs and TACs values^24^.

Landings of the three stocks are also constrained by a Minimum Conservation Reference Size regulation that was implemented for all stocks using values currently enforced in the studied fishery^28^. Likewise, from 2016, the Landings Obligation was implemented, with *de minimis* exemptions for hake and sole, depending on the year and the gear used to fish them^2,31–34^. See Supplementary Information’s sections 2.4 and A.3, Figure S2 and Table S10 for further details on these restrictions.

In response to the above management rules, a fishers’ behaviour algorithm has been developed to describe fishermen adaptation. Some *métiers* may be forbidden, depending on some conditions - the catch quota has been reached, the landings obligation is enforced - but also some values - the proportion of discarded catch, and also catch on previous years. Therefore fishermen change *métiers* within their strategy *métiers* set through a re-allocation of fishing effort to the latter set. This re-allocation aims to avoid quota overshooting. Further details about this algorithm are provided in the Supplementary Information’s sections 2.4 and A.3 and Figure S2.

### Calibration procedure

The model has been calibrated using two parameters (population accessibility and fishing target factor) involved in the catchability process (equation (21) in ISIS-Fish documentation^8^). The objective of the calibration is to reproduce the dynamics of catch over 2010–2012 at the species*métiers group scale, for each year or quarter depending on available data’s granularity. Calibration is sequentially performed: accessibility parameters for each population were estimated first followed by the target factors. The estimation of each parameter set (parameter type * population) combination was separated, and values were estimated jointly within each parameter set. To account for the specificity of each population model dynamics (global age-based for sole, spatial and size-based for hake, spatial, sex and size-based for Norway lobster), an objective function is defined for each population to calibrate their accessibility. More details on objective functions and procedures are provided in Supplementary Information’s section 2.5, as well as estimated values in Tables S17–S19.

#### Hake accessibility

The calibration for hake accessibility is based on a procedure developed for a former version of the database^25^. One parameter was estimated per quarter, all values being equal across length classes. The model outputs were fitted to hake catch observations in weight in the Bay of Biscay in 2010–2012 per length class.

#### Sole accessibility

One parameter was estimated per age class. The model outputs were fitted to WGBIE fishing mortality per age class for sole^27^ in 2010–2012.

#### Norway lobster accessibility

One parameter was calibrated per sex and length class. The model outputs were fitted to catch in numbers per length class and sex in 2010 per quarter provided by WGBIE.

#### About target factors

Target factors drive how the effort is distributed between populations, *métiers* and season*year combinations. They were split in 3 components: a fixed component derived from the SACROIS effort dataset analysis (Tables S25–S27), another fixed component driving inter-annual variations of fishing effort (Tables S22–S24), derived from catch observations, and finally an estimated component (Tables S28–S30), allowing to tune the model’s dynamics to observed catch. This section focuses on the estimation of the latter.

#### Hake target factors

20 parameters were defined, for each combination of the 5 groups of *métiers* (longliners, gillnetters, whitefish trawler (coastal), whitefish trawler (not coastal), Norway lobster trawler, see definition Table S8) and 4 quarters. We fitted the model’s outputs to the same data and with the same objective function as for hake’s accessibilities estimation.

#### Sole target factors

1 estimated component per group of *métiers* (gillnetters, Norway lobster trawlers and whitefish trawlers) and quarter. We fitted the model’s outputs to sole catch in weight on 2010–2012 for each *métier* and quarter.

#### Norway lobster target factors

1 estimated component per group of *métiers* (Norway lobster trawlers and whitefish trawlers). We fitted the model’s outputs to monthly Norway lobster landings data per length and sex class for 2010.

### Base simulation

The base simulation ran from January 2010 to December 2020 inclusive, with a monthly time step, using the database and parameters values described in this document. Several outputs of interest may be explored after a run: catch (discards and landings), as done in several figures in this paper, but also biomass (total biomass or mature biomass), fishing mortality values, or effort, all at a fine spatio-temporal scale.

## Data Records

All files of the database, as well as the companion document (a technical documentation), a base simulation and its outputs are stored on the SEANOE online repository^49^. The latter contains a read-me file, detailing what operations should be performed in order to use the database, re-run the base simulation and reproduce outputs. The following list describes the contents of each folder and file stored at the root of the repository:

- modelSkillAssessment folder: this folder contains scripts and other inputs that were used to produce the figures in this data descriptor, as well as other outputs. Inside this folder, figDiagnostics contains a Bay of Biscay shapefile, observationsSole and observationsLangoustine contain respectively data, scripts and outputs on observed sole catch and Norway lobster landings.
- export folder contains non Bay of Biscay catch outputs for hake (fields: monthly time step, year, size group, group of *métiers*, fraction (2 = landings, 1 = discards), catch in numbers)
- output folder contains several simulation outputs (see Table 1 for details).

**Table 1:** List of output files on the repository.

Name	Description	Fields
StrMetAfterChecks.csv	Effort re-allocation trace	monthly time step; *métier*; strategy; proportion of strategy effort after effort re-allocation
StrMetBeforeChecks.csv	Effort re-allocation trace	monthly time step; *métier* of origin; strategy; proportion of strategy effort before effort re-allocation; vector of *métiers* to which effort is re-allocated to; proportion of re-allocated effort to each of the latter *métiers*
TACvalues.csv	Simulated catch/landings quota values trace	year, population, quota in tons
TraceTarfs.csv	Changes done on target factors due to management rules	target factor name; original value, new value, ratio catch quota over expected catch/landings for hake, sole and Norway lobster
flagChecks.csv	*Métiers* flags trace	monthly time step; *métier*; flagged value
catchSpanishLGWithRule_0_3.csv	Spanish longliners and gillnetters hake catch outputs	monthly time step; unused field; fraction (0 = discards, 1 = landings); length group; catch in tons
catchSpanishLGNumWithRule_0_3.csv	Spanish longliners and gillnetters hake catch outputs	monthly time step; unused field; fraction (0 = discards, 1 = landings); length group; catch in numbers
catchSpanishLGNumSave_NoTAC2010.csv	Spanish longliners and gillnetters hake catch outputs	monthly time step; unused field; area; length group; catch in numbers
catchSpanishLGNumSaveBefore_NoTAC2010.zip	Spanish longliners and gillnetters hake catch outputs	monthly time step; unused field; area; *métier*; length group; catch in numbers
- simu folder is the output simulation folder, as created by ISIS-Fish in isis-fish-4/isis-database/simulations directory, and can be read by ISIS-Fish if placed again in that directory. Some files in resultExports (containing results to be plotted) were zipped to save space. See ISIS-Fish documentation about the contents of each file. The catch/landings/discards files do not contain information on Spanish longliners and gillnetters and on catch outside of the Bay of Biscay (see exports and output repertories).
- scripts folder contain scripts necessary to run the simulation, and are to be placed in specific folders (see README.MD on the repository).
- input folder: contains input files necessary to the simulation and the calibration procedure. See Table 2 for details.

**Table 2:** List of input files on the repository.

Name	Description	Fields
FiletsEspagnolsCaptureNombres2010.csv	Observed Spanish gillnetters hake catch in numbers for 2010	1 row per month, 1 column per length bin
PalangresEspagnolsCaptureNombres2010.csv	Observed Spanish longliners hake catch in numbers for 2010	1 row per month, 1 column per length bin
Ninit_hake_2010_AfterGrowth.csv	Hake abundance at the beginning of the initial time step	1 column per area; 1 row per length bin
Ninit_Sole_2010_WGBIE2017_1zone.csv	Sole abundance at the beginning of the initial time step	1 value per age bin
Ninit_nephrops_LANGOLF_without_migration_2010Corr.csv	Norway lobster abundance at the beginning of the initial time step	1 column per statistical rectangle and 1 row per length*sex bin
preSimulationScript.txt	Pre-simulation script to be copy-pasted into the pre-simulation section in the ISIS-Fish user interface	Java script
ratiosFiletsEspagnols1112.csv	Observed ratios of hake gillnetters catch (Spanish over French catch)	1 row per month and 1 column per size bin
ratiosPalangresEspagnols1112.csv	Observed ratios of hake longliners catch (Spanish over French catch)	1 row per month and 1 column per size bin
weight_at_length_hake.csv	Mean weight values in tons for each hake size bin	1 value per size bin
WeightingsLFD.csv	Objective function weightings for hake catch composition	quarter, group of *métiers*, fraction, weighting value
Weightingsweight.csv	Objective function weightings for hake catch in weight	quarter, group of *métiers*, fraction, weighting value
simulatingFleets folder	Observed hake catch in numbers for group of *métiers* out of the Bay of Biscay (group of *métiers* and fraction indicated in file name)	length bin in column, monthly time step from January 2010 to December 2016 in rows
- GdGMerluMSE_26Oct2018_1cm2cm10cm_ChangeMHake_Sole1Zone.zip: the database file to be loaded in ISIS-Fish (see ISIS-Fish online documentation). It contains most populations and exploitation assumptions.
- isisfish.bat a batch file launching ISIS-Fish, and saving a debug file.
- isis-fish-4-x-x-x.jar jar files, part of ISIS-Fish installation. It is recommended to use the most recent version.
- companion_document.pdf The companion document, a technical documentation providing further details on the database.

## Technical Validation

**Fig. 2:**
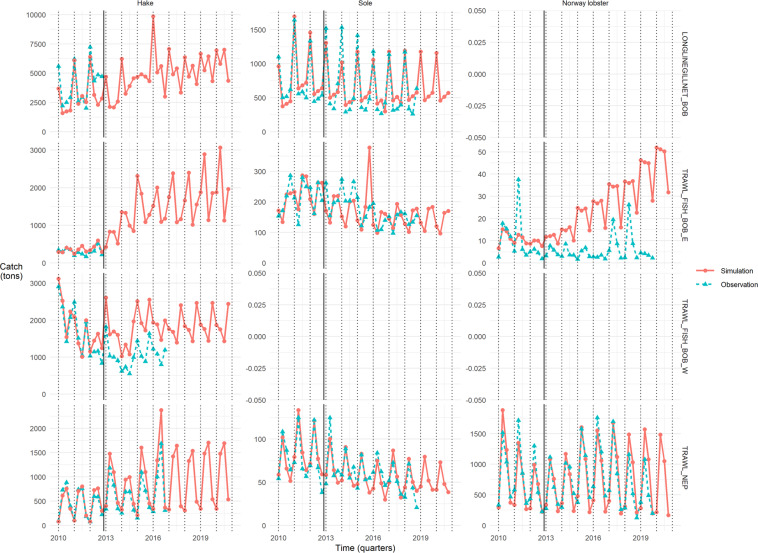
Quality of fit to catch (hake and sole) or landings (Norway lobster) observations for each population (facet columns) and group of *métiers* (facet rows) per quarter (x-axis). Catch/landings in tons are on the y-axis, simulated values in red and solid lines with round dots, observed values in blue and dashed lines with triangle dots. The black solid line delineates calibration (2010–2012) and simulation (2013–2020) periods; dotted black lines mark January months. Monthly values were aggregated by quarter to produce this figure.

The models ability to reproduce historical period dynamics is assessed using the quality of fit to catch (hake and sole) or landings (Norway lobster) observations. Quality of fit is shown for each quarter, population and group of *métiers* on Fig. 2, allowing to assess the inter-*métiers* and temporal (inter- and intra-year) variations of quality of fit; and to a lesser extent spatial variations, given that each *métier* is associated to a fishing area.

### Catch

#### Hake catch

In general, the model reproduces well the intra-annual catch patterns for each group of *métiers* when data was available, as well as the order of magnitude. However, on the time series, catch for Spanish trawlers is over-simulated once the calibration period is over, from 2013, by around 500–1000 tons each quarter. Likewise, Norway lobster trawlers catch is over-simulated from 2013, especially for quarters 2 and 3, by 250–500 tons each of these quarters. On the contrary, longliners and gillnetters catch is under-simulated for 2010 and 2012 by several thousands of tons, contrasting with the excellent quality of fit in 2011.

#### Sole catch

The model reproduces very well the order of magnitude of catch, as well as the intra-annual variations of catch for Norway lobsters trawlers. Regarding gillnetters (no longline catch), intra-annual variations are well caught on the calibration period, up to 2012, but less on the simulation period, especially on 2013–2015, when simulated values vary less than observed values. French whitefish trawlers catch is less well reproduced on the calibration period, with noticeable discrepancies on 2010–2011, and also strong under-simulations on 2014 (100 tons each quarter) and strong over-simulation on 2015 quarter 4.

Further comments on sole catch at the monthly scale are provided in Usage Notes section.

#### Norway lobster landings

The model reproduces very well the order of magnitude and intra-annual variations for Norway lobster trawlers, with a compromise between over-simulation for 2010 and under-simulation for 2011–2012 on the second quarter by a few hundreds of tons on the calibration period. Landings are again over-simulated at the end of time series, during 2018–2019 second and third quarters. The model was however not able to reproduce Norway lobster landings caught by French whitefish trawlers, with an overall over-simulation (except for a few peaks in observations) and lack of ability to catch the intra-annual trends. This is due to the smaller weight of the latter catch compared to Norway lobster trawlers catch in the objective function.

Further comments on Norway lobster landings at the monthly scale are provided in Usage Notes section.

### Spatial patterns

**Fig. 3:**
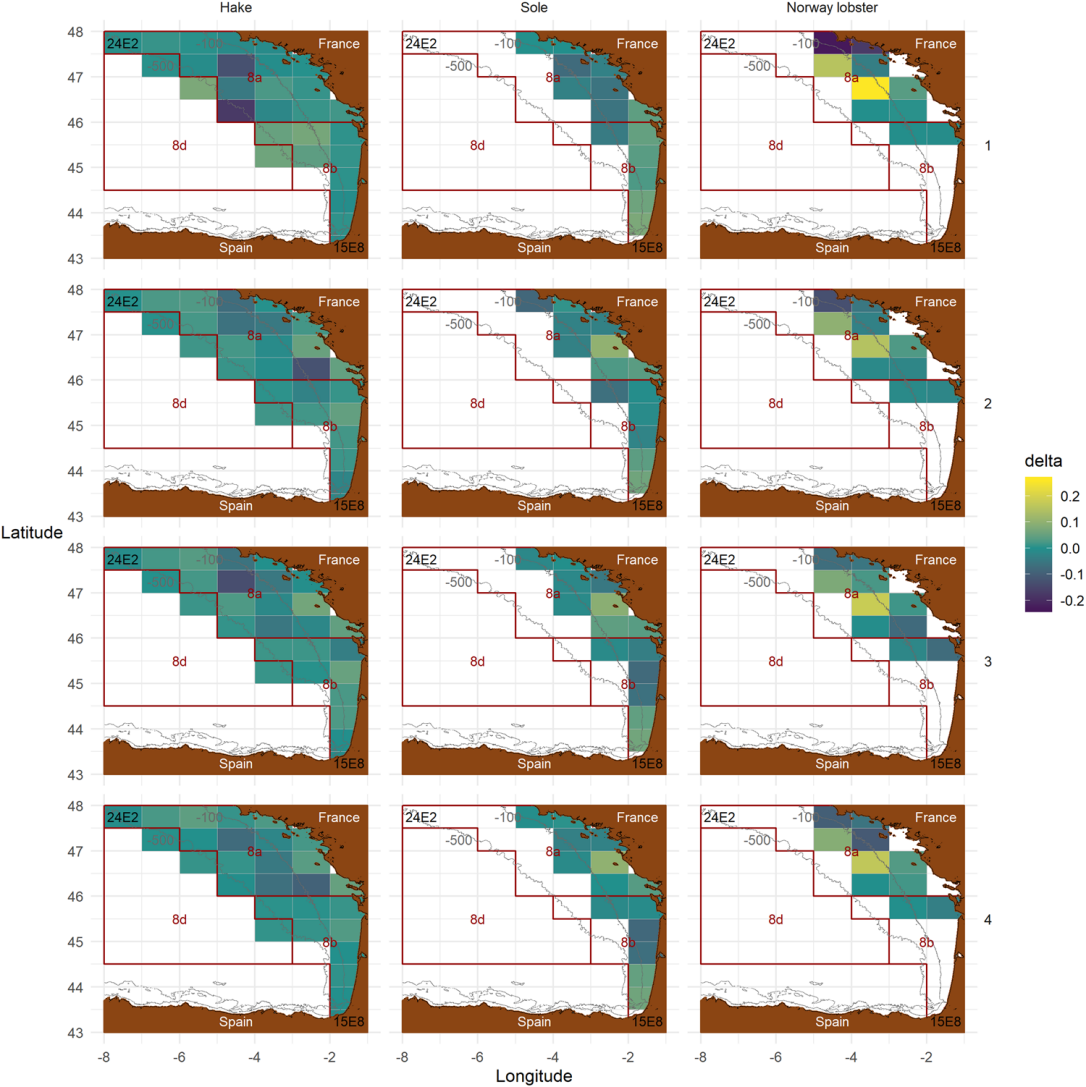
Map of discrepancies (*delta*) on French landings spatial distribution in 2010 for each population (facet column) and quarter (facet row) per ICES statistical rectangle (grey grid lines, 0.5° latitude by 1° longitude rectangles, most North-Western and South-Eastern rectangles 24E2 and 15E8 are indicated in black). *delta* is the difference of proportion of landings in a statistical rectangle between simulation and observations. A positive value (yellow) denotes an over-simulation, and a negative value (blue) an under-simulation. The study zone is bounded in ICES divisions 8a, 8b and a section of 8d (dark red boundaries). Isobaths −100m and −500m are indicated in grey, land is the brown area.

We assessed the model’s ability to reproduce historical spatial patterns by comparing observed and simulated spatial landings distribution for each population, ICES statistical rectangle and quarter on 2010 (Fig. 3). We derived discrepancies as follows for each population *pop*, quarter in 2010 *q* and statistical rectangle *rs* of the study area *BoB*:

1 $${\Delta }_{pop,q,rs}=\frac{{L}_{pop,q,rs}^{sim}}{{\sum }_{rs\in BoB}\,{L}_{pop,q,rs}^{sim}}-\frac{{L}_{pop,q,rs}^{obs}}{{\sum }_{rs\in BoB}\,{L}_{pop,q,rs}^{obs}}$$

with ${L}_{.,.,}^{sim}$ and ${L}_{.,.,}^{obs}$ respectively simulated and observed landings. A positive value denotes an over-simulation of landings distribution, a negative value an under-simulation, and value close or equal to 0 a good reproduction of the spatial distribution.

#### Hake landings

Discrepancies are stronger offshore during the first quarter, and then nearby the coast during the rest of year, following the intra-annual hake migrations. Several under- and over-simulation patches are noticed during quarter 1, with more over-simulation in the South; and during quarters 2–4, under-simulation in - roughly - the Great Mudbank, and over-simulation nearby the coasts, with some exceptions.

#### Sole landings

A quarter 1 pattern and quarters 2–4 patterns are also noticed. The former concentrates over-simulation on the most eastwards rectangles, and under-simulation in the most northwards and westwards rectangles; the latter shows a mix of under- and over-simulation over the coast.

#### Norway lobster landings

Roughly the same spatial pattern of discrepancies occurs during the 4 quarters, with the strongest discrepancies in the North of the presence area. The discrepancies slightly dampen along the year.

### Database uses and potential improvements

This database can be used to simulate fisheries dynamics over the historical data period. Its fit to catch and landings data is globally good, even though its performances are less good on the simulation period than on the calibration period. Better quality of fit could be achieved by redesigning the calibration procedure, using updated datasets, especially for hake, and by reviewing some assumptions on inter-annual exploitation dynamics.

Regarding spatial patterns, they were not explicitly accounted for during the calibration procedure, explaining the discrepancies between estimated and observed catch and landings spatial patterns. This could be improved by, among others, accounting for *métiers* instead of group of *métiers* in the objective function, since they bring spatial variability, or by directly accounting for the spatial pattern discrepancies in the calibration procedure.

## Usage Notes

### How to use the database

We invite users to follow the guidelines in the repository’s README.md file, explaining how to use the files and reproduce our work. We also strongly advise users to browse http://isis-fish.org, where can be found the latest files to install ISIS-Fish and its documentation.

### Fit to catch and landings observations at the monthly scale for sole and Norway lobster

**Fig. 4:**
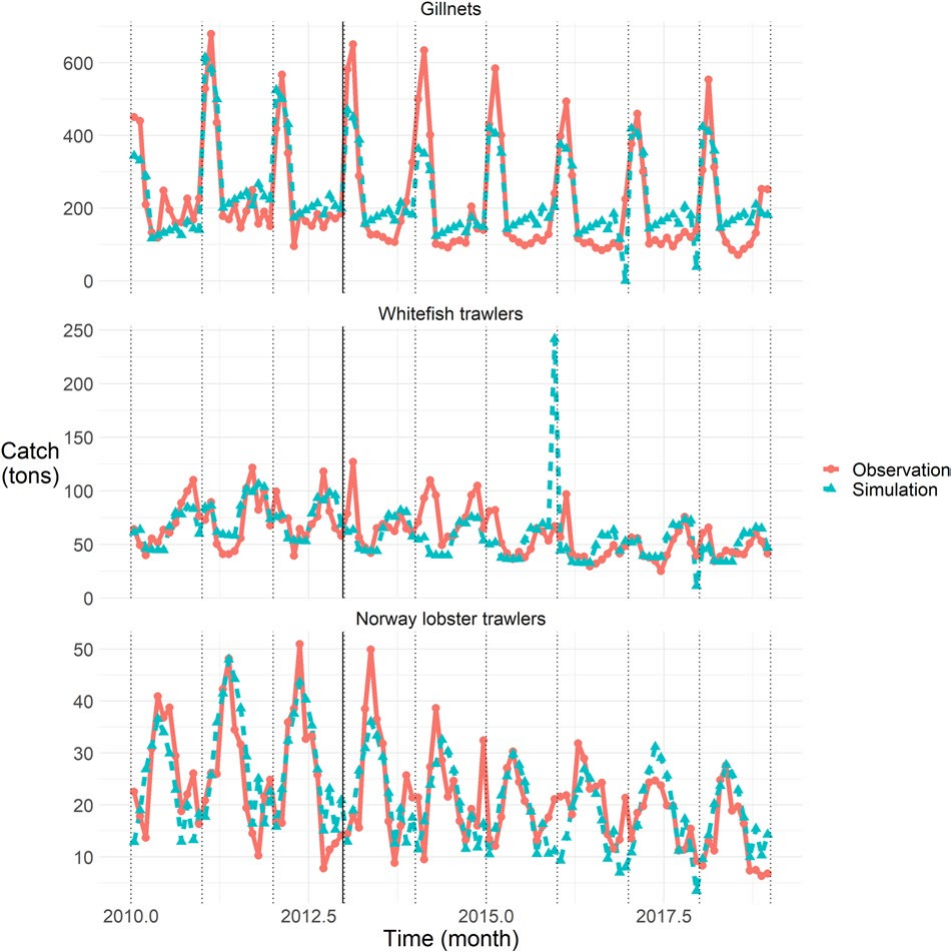
Quality of fit to sole catch observations for each group of *métiers* (facet) per month (x-axis); dotted black lines mark January months. The black solid line delineates calibration (2010–2012) and simulation (2013–2020) periods. Catch in tons is on the y-axis, simulated values in red and solid lines with round dots, observed values in blue and dashed lines with triangle dots.

Regarding fit to sole catch observations at the monthly scale, Fig. 4, we note, as on Fig. 2, that the order of magnitude and the intra-annual variations of catch are generally well reproduced; that the quality of fit for gillnetters is better on the calibration period (2010–2012) rather than the simulation period (from 2013); discrepancies on 2010–2011 and under-simulations in 2014 for whitefish trawlers. On top of that, we also notice that despite catching well the order of magnitude for whitefish trawlers, the model poorly reproduces the monthly catch pattern for that group of *métiers* on the time series, with numerous small discrepancies. This may be due to the temporal variations pattern in the observations, being less regular than for gillnetts or Norway lobster trawlers. We also notice that in December 2016 and 2017, the catch is under-simulated for gillnets, that in December 2017, the same goes for trawlers, and that in December 2015 the catch is over-simulated for whitefish trawlers: these are due to effort re-allocations from or to these *métiers*.

**Fig. 5:**
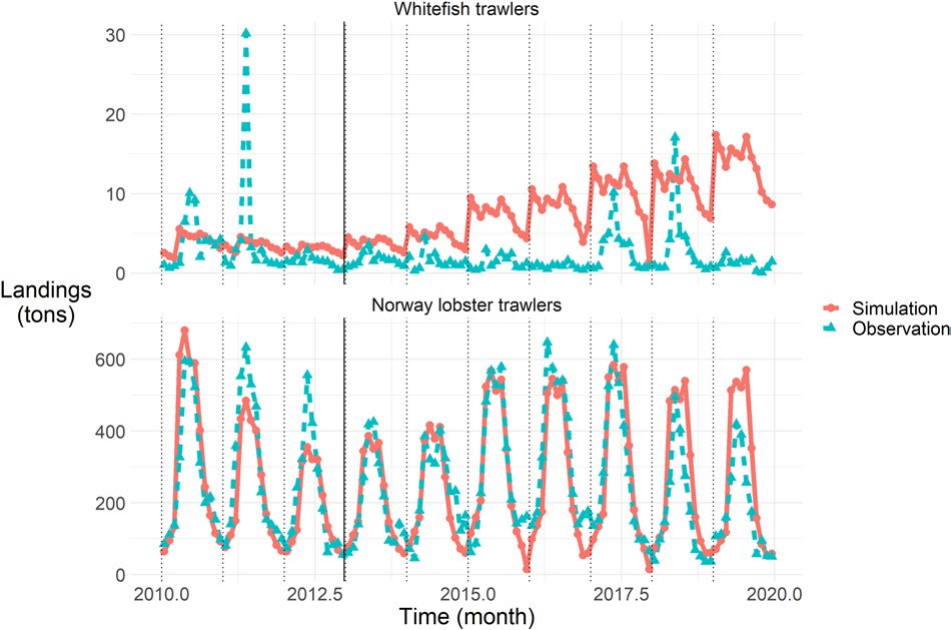
Quality of fit to Norway lobster landings observations for each group of *métiers* (facet) per month (x-axis); dotted black lines mark January months. The black solid line delineates calibration (2010–2012) and simulation (2013–2020) periods. Landings in tons are on the y-axis, simulated values in red and solid lines with round dots, observed values in blue and dashed lines with triangle dots.

Regarding fit to Norway lobster landings observations at the monthly scale, Fig. 5, we note, as on Fig. 2, that the order of magnitude and the intra-annual variations of landings are very well reproduced, with over- then under-simulation at the annual peak mid-year during the calibration period (2010–2012), and a trend to over-simulate at the end of the time series; the very poor reproduction of whitefish trawlers landings, in terms of order of magnitude (overall over-simulation) or monthly variations. On top of that, we notice here the under-simulation of whitefish trawlers landings in December 2017, and of Norway lobster trawlers in December 2015 and 2017, due, as for sole catch, to effort re-allocations from or to these *métiers*. Regarding whitefish trawlers, the hard-to-reproduce peaks in observations in 2011, 2017 and 2018 are also noticeable.

## Supplementary information

Companion document

## Data Availability

Code used to generate the Technical Validation and Usage Notes figures is provided on the repository^49^, as indicated in section Data Records. R scripts were ran with R version 3.6.0^50^, using packages *reshape2*^51^, *rgdal*^52^, *tidyverse*^53^ and *viridis*^54^.
