# Supplementary material for: Monthly spatial dynamics of the Bay of Biscay hake-sole-Norway lobster fishery: an ISIS-Fish database
Source: Sci Data. 2022 Jun 15;9:306. doi: 10.1038/s41597-022-01408-0 (PMC9200735; doi:10.1038/s41597-022-01408-0)
Supplement: Supplementary file 1 — Companion document [file 41597_2022_1408_MOESM1_ESM.pdf]

ISIS-Fish Bay of Biscay hake-sole-Norway lobster documentation

Audric Vigier

May 16, 2022

Contents

1 Generalities **2**

2 Detailed model description **2**

2.1 Time periods 2
2.2 Populations model 2
2.3 Exploitation model 8
2.4 Management 12
2.5 Calibration 14

A Additional information about ISIS-Fish parametrisation **17**

A.1 Biology 17
A.2 Exploitation 23
A.3 Management 36

1 Generalities

This is the companion document of the database stored on the following repository: https://doi.org/10.17882/86233.

This Bay of Biscay hake-sole-Norway lobster ISIS-Fish database was designed to improve our understanding of the above-mentioned fishery dynamics and help assessing current management tools to reach management objectives, on 2010-2020. This work has a strong emphasis on hake biology and its management.This database also includes some population and exploitation dynamics for hake in the Celtic Sea and a northern area (West of Scotland and North Sea), in order to account for the full northern hake stock area. However, given the poor performances of the model in these zones (unrealistic biomass collapse), dynamics of these zones were ignored when reading the base simulation outputs. Given our assumptions, this does not impact the Bay of Biscay dynamics. Further assumption writing work, as well as finer data is needed in order to properly use theses zones in the database.

2 Detailed model description

The model parametrisation is based on the one used for the projects Myfish (Worsøe Clausen et al., 2016), Benthis (Sala et al., 2014), and Coselmar (Pardo et al., 2017). It was altered to fit our modelling purposes on the hake-sole-Norway lobster mixed fisheries.

2.1 Time periods

Selected time period is 2010-2020. The base simulation was calibrated on 2010-2012 (see details section 2.5), validated on 2013-2016, the period of interest to assess current management (including landings obligation) being 2016-2020.

2.2 Populations model

Populations 3 populations/stocks are modelled: northern hake (*Merluccius merluccius*), common sole (*Solea solea*), and Norway lobster (*Nephrops norvegicus*).

Stocks structures

- Hake: length structure; 72 length classes; 39 1cm length classes, from [1;2[to [39;40[cm ; 30 2 cm length classes from [40;42[to [98;100[cm; and 3 10 cm length classes, [100;110[, [110;120[, [120;130+] cm (ICES, 2010). This structure is a compromise between the complexity needed to represent the structure of younger individuals (Drouineau (2008) among others; 2cm growth increments were too big; some individuals were not growing) and parcimony (2, then 10cm length bins are enough for bigger individuals).
- Sole: age structure; 7 age classes, from 2 to 8+ (ICES, 2013)
- Norway lobster: length-sex structure; 1 mixed recruitment class at 0cm; 33 male 2 carapace length mm length classes, from [10;12[to [72;74[carapace length mm; 23 female 2 carapace length mm length classes, from [10;12[to [52;54[carapace length mm

Zones Zones are defined basing on Vigier (2018) (with a change for sole):

- Hake: 6 zones, including 4 for the Bay of Biscay (presence, reproduction, recruitment, recruitment intermediate), 1 for the Celtic Sea and 1 for the northern area (West of Scotland + North Sea). See Vigier (2018)'s delineation figure S1a-S1d.
- Sole: only 1 zone (Vigier (2018)'s *a priori* zone, see figure S1e), given spatial artifacts were appearing in the modelling with 3 zones (all individuals ended up concentrated in only 1 zone; the complexity brought by the 3 zones did not allow us to get more knowledge on the fishery)

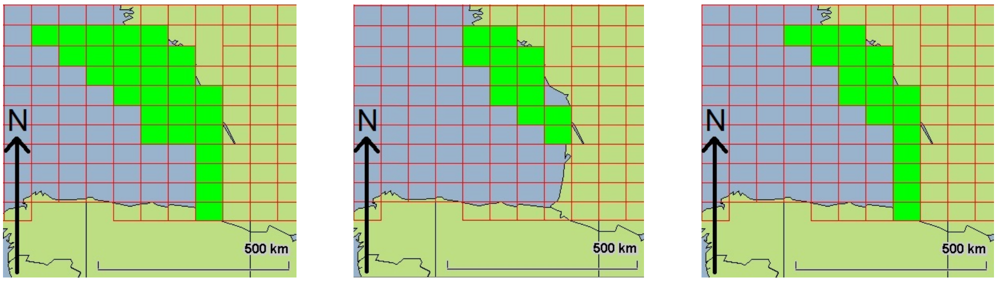

(a) Hake Bay of Biscay presence (b) Hake Bay of Biscay recruitment (c) Hake Bay of Biscay recruitment interim

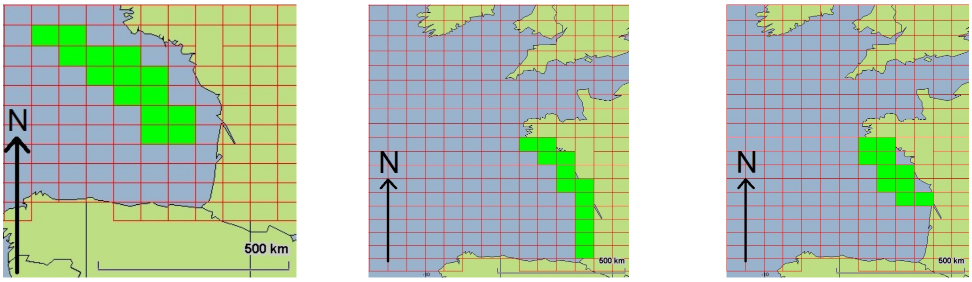

(d) Hake Bay of Biscay spawning (e) Sole presence (f) Norway lobster presence

Figure S1: Zones

- Norway lobster: the Great Mudbank (*la Grande Vasière*), see Vigier (2018)'s *a priori* zone, figure S1f

Seasons Same seasons as Vigier (2018), basing on maturity, movements and growth. For each stock*season*group combination, an accessibility parameter is defined and estimated in the calibration procedure (see section 2.5).

- Hake: 4 seasons/trimesters, at the beginning of which the following events occur. First trimester = aggregation of mature individuals on the shelf break ; Second trimester = Mature individuals begin to spread over the shelf; Third trimester = Mature individuals still present on the shelf break finish to spread over the shelf; Fourth trimester = migration from northern area to Celtic Sea (see areas paragraph "Zones"), also no hake recruitment during that season

- Sole: no seasons

- Norway lobster: 8 seasons are defined; 1/January, begins with the annual recruitment. Females are inside their burrows, less accessible; 2/ February-March females are inside their burrows, less accessible; 3/ April: Spring moulting, females are more accessible; 4/ May-June, females are more accessible; 5/ July-August, females are more accessible; 6/September, females are inside their burrows, less accessible; 7/ October: Autumn moulting only for immature females and all males, females are inside their burrows, less accessible; 8/ November-December, females are inside their burrows, less accessible (Conan, 1975)

Natural mortality

- Hake: constant, M=0.5, instead of the more commonly used M=0.4 (ICES, 2014a). We choosed M=0.5 given that the biomass, and then catch in Bay of Biscay had a too string increase across time steps with a lower M. A higher M value would have worsened too much the abundance collapse pattern in Celtic sea and northern area. M=0.5 also proved to provide the best fit to hake catch, without trtiggering an unrealistic discarding pattern.

- Sole: constant, M=0.1 (ICES, 2013)

Age	2	3	4	5	6	7	≥ 8
Weight (kg)	0.191	0.220	0.290	0.360	0.442	0.509	0.820

Table S1: Sole mean weight-at-ages

Age	≤ 1	2	3	4	≥ 5
Proportion	0	0.32	0.83	0.97	1

Table S2: Proportions of mature individuals in each sole age class

Year	2010	2011	2012	2013	2014	≥2015
Recruits (thousands)	277067	359154	359154	333712	224523	359154

Zone-season \ Year	2010	2011	2012	2013	2014	≥2015
Bay of Biscay	0.6336002	0.6873937	0.6873937	0.6873937	0.6873937	0.6873937
Celtic Sea	0.2650488	0.1999135	0.1999135	0.1999135	0.1999135	0.1999135
Northern area	0.101351	0.1126927	0.1126927	0.1126927	0.1126927	0.1126927
season 1	0.1301261	0.2513410	0.2513410	0.3768482	0.2595000	0.2513410
season 2	0.5878643	0.408250	0.408250	0.3001289	0.5180656	0.408250
season 3	0.2820096	0.3404011	0.3404011	0.3230229	0.2224344	0.3404011

Table S3: Hake global recruitment ans its spatial and seasonal allocation on 2010-2020

- Norway lobster: M=0.3 for all males and mature females (strictly over 22mm), M=0.2 for all immature females (under or equals 22mm) (Morizur, 1982)

Weight-at-class

- Hake: $W = a * l^b$, with W the weight in kg, l the mid of a size bin in cm, $a = 5.13 * 10^{-6}$ and $b = 3.074$ (ICES, 1991)
- Sole: see table S1 (ICES, 2013)
- Norway lobster: $W = a * l^b$, with W the weight in kg, l the mid of a size bin in cm. Males: $a = 0.39$ and $b = 3.18$. Females: $a = 0.81$ and $b = 2.97$ (Conan, 1978)

Maturity

- Hake: Maturity ogive from ICES (2010) $M_l = \frac{1}{1+e^{-0.2(l-42.85)}}$, with M_l the proportion of mature individuals inside a size class, and l the mid-size class in cm.
- Sole: see table S2 (ICES, 2013)
- Norway lobster: male individuals are considered mature from length class [26;28[carapace length mm; [22;24[for females (ICES, 2016)

Recruitment All individuals recruit at the smallest size bin of their stock.

- Hake: at the beginning of each month in January-September. Recruitment is allocated in each zone (Bay of Biscay recruitment zone, Celtic Sea and northern area) and month basing on ICES (2017) and Vigier et al. (2018) estimates. It is then deterministic and independent from biomass variations in ISIS-Fish. Individuals recruited at the beginning of year y, month m and zone z $R_{y,m,z}$ are derived with the following formula:

$$R_{y,m,z} = \frac{1}{3} R_y * p_{s,y} * p_{z,y} \quad (1)$$

Year	2010	2011	2012	2013	2014	2015	2016
Recruits (thousands)	24110	20305	12946	13080	15751	20152	18246

Table S4: Sole recruits in thousands estimated by ICES (2017)

with R_y the global annual recruitment for year y, $p_{s,y}$ the proportion allocated to the season s (to which the month m belongs) at year y, and $p_{z,y}$ the proportion allocated to the zone z. All values are summarized in table S3.

- R_y estimates: 2010 Vigier et al. (2018)'s estimate; 2011-2012 are the 2 last years of Vigier et al. (2018), so we take their geometric mean on 1978-2010 instead $R_{gm} = \frac{1}{33}\sum_{y=1978}^{2010} ln(R_y^{\text{Vigier et al. (2018)}})$; 2013-2014 uses information provided by ICES (2017)'s on annual recruitment variation relative to 2010: $R_y = R_{2010}^{\text{Vigier et al. (2018)}} * \frac{R_y^{\text{ICES (2017)}}}{R_{2010}^{\text{ICES (2017)}}}$. From 2015, we considered no information is available on recruitment and are using estimates for 2011-2012.
- $p_{s,y}$ estimates: for 2010, we use Vigier et al. (2018)'s estimates of seasonal allocation of recruitment. 2011-2012: with a reasoning similar to the one for annual recruitment, we use proportions on geometric means for each season $R_{gm,s} = \frac{1}{33}\sum_{y=1978}^{2010} ln(R_{y,s}^{\text{Vigier et al. (2018)}})$, which give $p_{gm,s} = \frac{R_{gm,s}}{\sum_{s\in S} R_{gm,s}}$. 2013-2014: we use ICES (2017)'s estimates. From 2015, we considered no information is available on recruitment and are using estimates for 2011-2012.
- $p_{z,y}$ estimates: for 2010, we use Vigier et al. (2018)'s estimates of spatial allocation of recruitment. 2011-2020: with a reasoning similar to the one for annual recruitment, we use proportions on geometric means for each zone $R_{gm,z} = \frac{1}{33}\sum_{y=1978}^{2010} ln(R_{y,z}^{\text{Vigier et al. (2018)}})$, which give $p_{gm,z} = \frac{R_{gm,z}}{\sum_{z\in Z} R_{gm,z}}$.

• Sole: at the beginning of each year. Individuals recruit at age 2, ages 0 and 1 not being modelled. On 2010-2016, recruitment is forced to equal ICES (2017) estimates (see table S4). A Hockey Stock relationship is then used for 2017-2020:

$$R_y = \begin{cases} 1.9SSB & , SSB < 9596t \\ 1.8247 * 10^7, & SSB \geq 9596t \end{cases} \tag{2}$$

• Norway lobster: recruitment is annual, modelled with a Beverton-Holt relationship: $R_y = \frac{\alpha SSB_y}{\beta + SSB_y}$, with SSB_y the mature female SSB in kg at the beginning of year y, R_y the recruitment in numbers, $\alpha = 1220695327.3$ and $\beta = 3866855.3$ (ICES, 2014b)

Growth

• Hake: growth increments. Growth modelling is based on ICES (2010)'s assumptions, and is similar to Drouineau (2008); Vigier (2018), except for differences with Vigier et al. (2018) to account for the different widths of size bins:

- A linear function between ages 0 and 0.75

$$l(t) = \frac{t}{9} * \bar{L_{0.75}} \tag{3}$$

with t the age in months, $\bar{L_{0.75}}$ the mean length at age 0.75 year.

– von Bertalanffy (von Bertalanffy, 1938) from age 0.75 :

$$l(t) = L_\infty(1 - e^{-K(t-t_0)}) \tag{4}$$

with L_∞ the asymptotic length , K a growth rate, and t_0 an "artifact" coefficient supposed to represent the age in months when mean size equals 0. t_0 is the solution of $\bar{L_{0.75}} = L_\infty(1 - e^{-K(x-0.75)})$. Following coefficients were estimated by ICES (2010) : $L_\infty = 130\ cm$, $K = 0.177319\ an^{-1}$, $\bar{L_{0.75}} = 15.8392\ cm$. From these comes that $t_0 \simeq 0.01727\ an$.

ISIS-Fish structure being in length only, it is not possible to use directly that relationship, function of the age. Growth is then modelled with:

$$N_{t+1} = GN_t \tag{5}$$

with N_t the vector if abundance for all length classes at time step t, and G the transition matrix indicating the probability to go from a length class to another one. G is constant across time steps. Growth increments approach was used to derive G. A mean growth increment for a fish of size l $\Delta_l(l)$ in time interval Δ_t is linearly defined before $\bar{L_{0.75}}$. After that length, it is defined with Fabens (1965)'s von Bertalanffy reformulation:

$$\Delta_l(l) = \begin{cases} \frac{\bar{L_{0.75}}}{9} & , \quad l < \bar{L_{0.75}} \\ (L_\infty - l) * (1 - e^{-K\Delta_t}) & , \quad l \geq \bar{L_{0.75}} \end{cases} \tag{6}$$

Interindividual growth variability is modelled with Drouineau (2008)'s assumptions. Growth increments during a time step are X random variables, of which the mean is derived using von Bertalanffy equation, and of which variance scales to the square of the mean. Then:

$$E(X/l) = \Delta_l(l) \tag{7}$$

$$V(X/l) = \frac{1}{\alpha} * E(X/l)^2 \tag{8}$$

with α a variance constant (strictly superior to 0). We assume that $CV = 1$, which gives $\alpha = 1$.

Finally, it is assumed that growth increments are gamma distributed: $X \sim G(\alpha, \beta_l)$, of which density function is :

$$f_l(x) = \frac{1}{\Gamma(\alpha)\beta_l{}^\alpha} x^{\alpha-1} e^{-\frac{x}{\beta_l}} \tag{9}$$

with $\beta_l = \dfrac{E(X/l)}{\alpha}$, Γ Euler's gamma fucntion: $\Gamma(x) = \int\limits_0^{+\infty} u^{x-1}e^{-u}du$.

The transition matrix G is then derived as follows:

$$g_{ij}^{temp} = \begin{cases} \frac{1}{U_i - L_i} \int\limits_{L_i}^{U_i} (\int\limits_{L_j - y}^{U_j - y} f_{m_i}(x)dx)dy, \ \forall j > i \\ \frac{1}{U_i - L_i} \int\limits_{L_i}^{U_i} (\int\limits_{0}^{U_j - y} f_{m_i}(x)dx)dy, \quad j = i \\ 0 \qquad , \forall j < i \end{cases} \tag{10}$$

with L the set of length classes, g_{ij}^{temp} the probability to go from length class i to class j during time interval Δ_t, L_j and U_j respectively lower and upper bounds of length class j, L_i and U_i those of class i and $m_i = \dfrac{L_i + U_i}{2}$.

However, given that length classes widths were different, the derived probabilities lead to a mean increment being too high just before a change of length class width. g^{temp} are then "corrected", by increasing the probability of not growing and decreasing the probability of growing:

$$g_{ij} = \begin{cases} g_{ij}^{temp} * \frac{\Delta_l(i)}{\sum\limits_i g_{ik}^{temp} * X_{ik}} & , \forall j \neq i \\ \\ g_{ij}^{temp} - (\sum\limits_{i \neq k} g_{ik} - \sum\limits_{i \neq k} g_{ik}^{temp}), & \forall j = i \end{cases} \tag{11}$$

with X_{ik} the actual increment when going from class i to class k.

G is then :

$$G = \begin{pmatrix} g_{11} & g_{12} & \cdots & g_{1n} \\ 0 & g_{22} & \cdots & g_{2n} \\ \vdots & \ddots & \ddots & \vdots \\ 0 & \ldots & 0 & g_{nn} \end{pmatrix} \tag{12}$$

with n the number of length bins.

- Sole: individuals get older
- Norway lobster: growth is modelled by growth increments, similarly to hake, except $g_{ij} = g_{ij}^{temp}$, since the width of length classes is constant for Norway lobster. One matrix is derived per sex, and to mime the moulting phenomenon, growth is possible only in April (all Norway lobster) and October (males and immature females only). Parameters are : $L_\infty = 76mm$, $K = 0.14 year^{-1}$ (males and immature females); $L_\infty = 56mm$, $K = 0.11 year^{-1}$ (mature females) (Conan, 1978)

Migrations Defined only for hake, and summarized in table S11. 2 sets of migrations are defined:

- One related to spawning and recruitment in the Bay of Biscay: aggregation at the beginning of the year on the shelf break to spawn, and then dispersion on the shelf (Casey and Pereiro, 1995; Guichet, 1996; Poulard, 2001; Alvarez et al., 2004; Woillez et al., 2007). Aggregation occurs at the beginning of January only for mature individuals (except first time step, as mature individuals are already in the spawning zone), and dispersion occurs progressively in April, then July (all individuals that did not spread yet spread). Also, from age 1 (around 20cm), individuals in recruitment zone spread in interim recruitment zone, to model a diffusion towards areas neighbouring the nursery area, at the beginning of each time step.
- One related to an inter-box movement, from the northern area to the Celtic Sea, estimated by Vigier et al. (2018)

Accessibility One accessibility parameter per stock*group*season is defined, and estimates with the estimation procedure described later (see section 2.5).

Abundance at initial step For each stock, abundance is provided for the beginning of January 2010 for each zone and class prior to the simulation:

- Hake: Based on Vigier et al. (2018)'s spatial estimates. See table S13 for values. Intra-Bay of Biscay allocation is not provided by Vigier et al. (2018); we assumed the following:

Gear	Catchable species	Standardisation coefficient
Trawl	Hake, sole, Norway lobster	1.39
Baka trawl	Hake	7.5
VHVO trawl	Hake	6.0
Norway lobster trawl	Hake, sole, Norway lobster	1.4
Gillnets	Hake, sole	0.37
Longlines	Hake, sole	1.15

Table S5: Modelled gears in the Bay of Biscay, catchable species, and standardisation coefficients

Gear	Function type	Parameters values
Twin trawl	Logistic	$p_1 = 27.0983$; $p_2 = 3.64362$
Norway lobster twin trawl	Double normal	$p_1 = 16.1476$; $p_2 = -14.0259$; $p_3 = 3.1092$; $p_4 = 4.89597$; $p_5 = -999$; $p_6 = -2.27506$
Spanish trawls	Double normal	$p_1 = 19.9193$; $p_2 = -1.59628$; $p_3 = 3.51534$; $p_4 = 4.10591$; $p_5 = -13.3064$; $p_6 = -0.709587$
Gillnets and longlines	Double normal	$p_1 = 71.1087$; $p_2 = -3$; $p_3 = 5.92049$; $p_4 = 2.98182$; $p_5 = -999$; $p_6 = -0.867108$

Table S6: Length selectivities modelling for each gear for hake. See equations (18) and (19) for functions formulae

- Spawning area: all matures individuals over 20cm are assumed to be in that area. For each bin, the number of mature individuals is derived with the maturity ogive (see above)
- Recruitment area; all individuals of which length is strictly under 20 cm are assumed to be less then 1 year old and to be recruits, so they are all in this zone
- Interim recruitment area: all individuals that have not been allocated to one of the 2 areas above
- Presence area: empty

Finally, since growth is not modelled for the first time step in ISIS-Fish, the transition matrix has been applied to all zones for hake.

- Sole: ICES (2017)'s estimates. See estimates in table S14.
- Norway lobster: ICES (2014b) and LANGOLF marine survey estimates were used, as detailed in Vigier (2018)'s appendix C.3. See estimates in table S16.

2.3 Exploitation model

Gears Gears, their set of species, and their standardisation coefficients are introduced in table S5. Coefficients values were derived in Marchal (2005)

Selectivities

- Hake: all hake selectivities were derived by Vigier et al. (2018). See table S6 for types of formula and parameter values for each gear. 2 types of formula were used (Methot and Wetzel, 2013), see equations (18) and (19).
- Sole: trawls, Norway lobster trawls and nets catch sole with a constant selectivity of 1. Hence, accessibility is mix of accessibility (catchability *sensu* Seber (1982)) and selectivity in the case of sole
- Norway lobster: trawls and Norway lobster trawls have the same selectivity function when catching Norway lobster (ICES, 2006):

$$Sel(l) = \frac{1}{1 + e^{-0.32*(l-22.36)}} \tag{13}$$

Gear	Function type	Parameters values
Twin trawl	Constant by intervals	$0\ ,\forall l < 27cm;\ 1\ ,\forall l \geq 27cm$
Norway lobster twin trawl	Logistic	$p_1 = 29.8012;\ p_2 = 2.11801$
Spanish trawls	Logistic	$p_1 = 28.3948;\ p_2 = 1.21606$
Gillnets and longlines	Constant by intervals	$0\ ,\forall l < 27cm;\ 1\ ,\forall l \geq 27cm$

Table S7: Length retentions modelling for each gear for hake. See equations (18) and (19) for functions formulae

Retentions Retention is the proportion of catch being landed, in $[0;1]$:

- Hake: all hake retentions were derived by Vigier et al. (2018). See table S7 for types of formula and parameter values for each gear (only for discarding gears). On top of that, all fleets, including the catch-forced ones, discard their undersized individuals (hake size < 27cm)
- Sole is assumed not to be discarded, except because of management constraints. Hence, retention is constant and equals 1 for sole (undersized soles are not modelled)
- Norway lobster is assumed not to be discarded, except because of management constraints. All under-sized Norway lobsters are discarded (size < 26mm CL)

Métiers Here, a *métier* is the combination of a gear, a zone and a mix of species. The set of modelled *métiers* was defined:

- French Bay of Biscay *métiers* : by analysing French effort data for 2010 (SACROIS database), following Deporte et al. (2012)'s methodology
- Spanish Bay of Biscay trawlers: definition from TECTAC project (Marchal, 2005)

Métiers main features are summarized in table S8 and figure S3. For Spanish longliners and gillnetters and non Bay of Biscay *métiers* , see paragraph "Other fleets".

Fleets Fleets were defined with the same data and methodology as for *métiers* . See a summary of their features in table S9. For Spanish longliners and gillnetters and non Bay of Biscay fleets, see paragraph "Other fleets".

Strategies Strategies were defined with the same data and methodology as for *métiers* . See a summary of their features in tables S9, S20 and S21. No strategy was defined for Spanish longliners and gillnetters and non Bay of Biscay fleets.

Bay of Biscay inter-annual fleets dynamics These are necessary to account for catch temporal variations, and are modelled through the parameter $VarsEff_{met,s,y,sp}$, added to Pelletier et al. (2009)'s equation (21) as a multiplicative factor on effort. It is derived as following:

- 2010-2016, a ratio of observations for year y over observations for 2010 is used (ICES, 2017). Hake, one per *métier* *season*year: $VarsEff_{met,s,y,sp} = \frac{C^{obs}_{met,s,y}}{C^{obs}_{met,s,2010}}$; Sole, one per *métier* *year $VarsEff_{met,s,y,sp} = \frac{C^{obs}_{met,y}}{C^{obs}_{met,2010}}$; Norway lobster, one per year $VarsEff_{met,s,y,sp} = \frac{C^{obs}_{y}}{C^{obs}_{2010}}$
- 2017-2020: at the time of the writing of these assumptions, more recent data was not available, and ratios were deduced from trends on 2014-2016. A linear model was fitted on ratios deduced earlier on 2014-2016. If a significant trend was identified (Hake TRAWLOTH s2;4, LONGLINE and GILLNET s2;3, all sole and Norway lobster *métiers*), the slope was used to deduce 2017-2020 ratios (the slope was halved for hake TRAWLOTH and sole and Norway lobster *métiers* to avoid unrealistic high values of effort). Otherwise, 2016 ratios were used. See tables S22, S23, and S24 for values.

Métier	Stock	Gear	Matching super-*métier*
Metier_ChalutMixte_NordPC<br>Metier_ChaluMixte_InterC<br>Metier_ChalutBenth_NordAPC<br>Metier_ChalutBenth_NordC<br>Metier_ChalutBenth_APCS<br>Metier_ChalutSole_InterC<br>Metier_ChalutSole_NordCet<br>Metier_ChalutSole_InterSudC<br>Metier_ChalutMixte_NordC<br>Metier_ChalutMixte_APCS	HSN	Trawl	TRAWL_FISH_BOB_E
Metier_Lang_InterC<br>Metier_Lang_NordPC<br>Metier_Lang_InterPC	HSN	Norway lobster trawl	TRAWL_NEP
OBTS_VIIIabd	H	Baka	TRAWL_FISH_BOB_W
PTBV_VIIIabd	H	VHVO	TRAWL_FISH_BOB_W
Metier_FiletSole_NordC<br>Metier_FiletSole_NordIntPC<br>Metier_FiletSole_InterSudPC<br>Metier_FiletSole_InterC<br>Metier_FiletMerlu_NordC<br>Metier_FiletMerlu_NordPC<br>Metier_FiletMixte_NordPC<br>Metier_FiletMixte_NordC<br>Metier_FiletMixte_NordInterPC<br>Metier_FiletMixte_InterSudC<br>Metier_FiletMIxte_InterC<br>Metier_FiletMerlu_InterSudAPC<br>Metier_FiletMerlu_InterSudC<br>Metier_FiletMerlu_InterSudPC	HS	Gillnet	GILLNET_BOB
Metier_PalangreMerlu_InterSudAC<br>Metier_PalangreMixte_NordC<br>Metier_PalangreMixte_InterC<br>Metier_PalangreMerlu_InterSudC	H	Longline	LONGLINE_BOB

Table S8: Modelled *métiers* in the Bay of Biscay, catchable stocks (H: hake, S: sole, N: Norway lobster), gears, and matching super-*métier* . Super-*métiers* are clusters of *métiers* matching Vigier et al. (2018)'s *métiers* . All *métiers* fish year-round.

Fleet	Strategy	Ratio	Vessel type	Number of vessels
ON_30_39	SpainMainMegrimAnglerfishON_30_39<br>SpainMainHakeON_30_39	0.66<br>0.33	Spanish<br>30m	36
ON_20_29	SpainMainMegrimAnglerfishON_20_29<br>SpainMainHakeON_20_29	0.73<br>0.27	Spanish<br>24m	15
PA_30_39	SpainMainHakePA_30_39<br>SpainMainMegrimAnglerfishPA_30_39	0.89<br>0.11	Spanish<br>30m	18
GdG_Nord_Plus18m	STR_GdG_Nord_plus18m	1	French<br>24m	85
GdG_Sud_1218m	STR_GdG_Sud_1218m_O<br>STR_GdG_Sud_1218m_G	0.54<br>0.46	French<br>15m	68
GdG_Sud_Plus18m	STR_GdG_Sud_plus18m_O<br>STR_GdG_Sud_plus18m_G	0.38<br>0.62	French<br>24m	78
GdG_Nord_1218m	STR_GdG_Nord_1218m_O<br>STR_GdG_Nord_1218m_G	0.85<br>0.15	French<br>15m	133
GdG_Sud_moins12m	STR_GdG_Sud_moins12m_G-L-O<br>STR_GdG_Sud_moins12m_G<br>STR_GdG_Sud_moins12m_O<br>STR_GdG_Sud_moins12m_L	0.1<br>0.39<br>0.43<br>0.07	French<br>10m	177
GdG_Nord_moins12m	STR_GdG_Nord_moins12m_G-L<br>STR_GdG_Nord_moins12m_G<br>STR_GdG_Nord_moins12m_O	0.05<br>0.37<br>0.58	French<br>10m	93

Table S9: Modelled fleets and strategies in the Bay of Biscay, proportions of each fleet following a strategy, vessel types (including length in m) and number of vessels.

Other fleets Spanish longliners and gillnetters and non Bay of Biscay *métiers* could not be explicitly described by effort due to lack of data. They are instead modelled by a catch in numbers, derived prior to the simulation, to be removed from abundance (in the limit of available individuals) in the post-action of each time step. They catch only hake. See Vigier (2018)'s appendix C.2 for a comprehensive description of these *métiers* .

That type of modelling for these *métiers* , combined to the inability of a size-structured model to follow hake cohorts, is thought to be what causes the abundance collapse pattern in the Celtic Sea and the northern area.

Target factors Target factors are multiplicative coefficients in Pelletier et al. (2009)'s eq. (21). In this parametrisation, target factors are the products of 2 components:

- A fixed component, for each species**métier* *season, derived during SACROIS French effort data analysis (which is the product of a *métier* component and a season component). See tables S25, S26, and S27 for estimated values
- An estimated component, for each species*gear*season, derived within the estimation procedure (see section 2.5)

Year	2013	2014	2015	2016	2017	2018	2019	2020
Hake Bay of Biscay	25970	30610	33977	**40393**	**44808**	**42460**	**52118**	**50441**
Sole Bay of Biscay	4100	3800	3800	**3420**	**3420**	**3621**	**3872**	**3666**
Norway lobster Bay of Biscay	3899	3899	3899	3899	4160	3614	3878	3886

Table S10: Historical TAL/TAC (bold font) values in tons for Bay of Biscay hake, sole and Norway lobster

2.4 Management

TAL/TAC management All stocks are managed by TALs/TACs, which values are the ones historically implemented (see table S10). Their values are considered as TALs until 2015, and as TACs from 2016 (except for Norway lobster, not being under the landings obligation):

- 2010-2012: no landings limitation is implemented, since this period is also the calibration period. It was chosen to favour a better parametrisation, allowing for more reliable dynamics on the following years of the time series, rather than a full compliance to 2010-2012 TALs that could have compromised the dynamics from 2013, through ctach over-simulation

- From 2013: annual TALs/TACs are implemented for each stock. Given hake dynamics modelling, only Bay of Biscay hake TAL/TAC is modelled as a management constraint. From 2017, when observations out of the Bay of Biscay are no more available, the quantity of hake caught in these areas is scaled on the Bay of Biscay TAC, using proportions in compliance with relative stability (see Vigier (2018) appendix D.2)

MSY transition scenarios from 2016 to 2020 were considered, but had to be dropped out because of our unability to properly estimate F_{MSY} reference point for hake (collapse pattern out of the Bay of Biscay).

Minimum Landing Size All catch has to be discarded (unless landing obligation is constraining) when individuals are smaller than the following lengths:

- Hake: 27cm

- Sole: undersized individuals are not modelled (under age 2)

- Norway lobster: 26 mm CL

Landings obligation Landings obligation is implemented form 2016 for hake and sole, with *de minimis* exemptions for some *métiers* catching them:

- Hake trawlers only: 7% in 2016-2017, 6% in 2018-2019, 5% in 2020

- Hake longliners and gillnetters: no exemption

- Sole trawl *métiers* : 5% ; net *métiers* : 3%

Fishers behaviour Fishers behaviour is modelled by 3 main sets of assumptions: 1/ their reaction to the mix of TAC/TAL, minimum landing size and landing obligation management 2/ Adaptation of fishing effort when TAC/TAL is nearly reached 3/ Adaptation of fishing effort to the current TAL/TAC to spread the catches over the whole year.

1/ At the beginning of a time step, a *métier* is allocated to a category according to the decision tree figure S2. That tree is explored for each of the 3 stocks, and the highest category number among the species caught by the *métier* is allocated to the *métier* . Depending on the category (and "must land" flag for hake), the *métier* :

- Category 0: operates without constraint

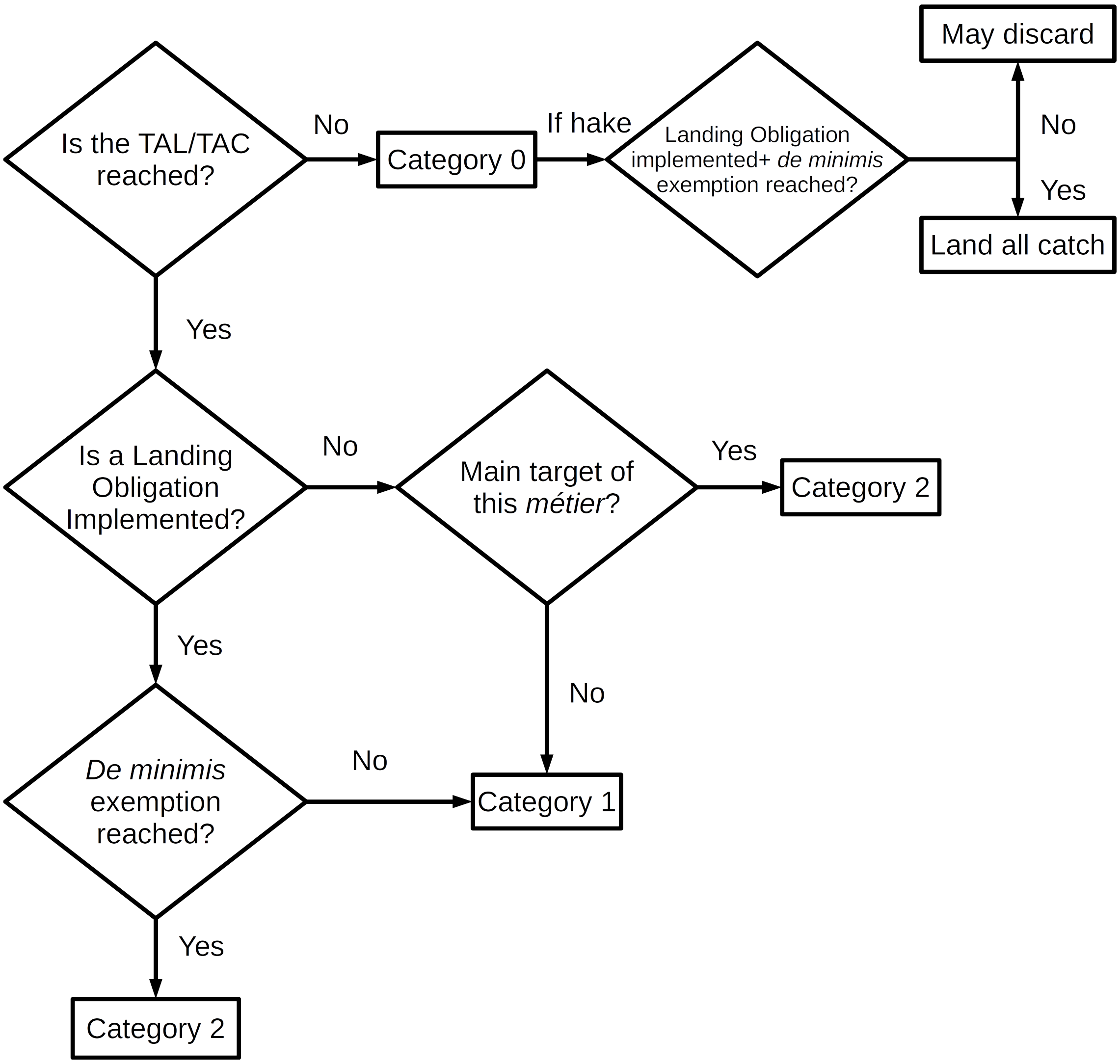

Figure S2: Decision tree to allocate *métiers* into categories. The tree is explored by each species**métier* combination; each *métier* is then allocated to the highest category the species it can catch reached

- Category 1: can only discard its catch for species whose TAL/TAC is reached; no constraint on other species
- Category 2: is forbidden, and its effort is re-distributed to other *métiers* (see below for details on re-allocation)
- Hake case: if the TAC is not reached by the landings obligation is implemented, the *métier* has to land its catch if the *de minimis* exemption is reached, operate without constraint otherwise

Category 2 *métiers* effort is then re-allocated in the following order, in each strategy:

- to non-forbidden *métiers* among the same strategy and using the same gear
- if said *métiers* do not exist, to other *métiers* in the same strategy having a month-strategy-*métier* proportion (see table S21) strictly over 0
- if said *métiers* do not exist, to other *métiers* in the same strategy having a month-strategy-*métier* proportion (see table S21) equal to 0
- if said *métiers* do not exist, the effort is not re-allocated

In all cases, effort for the forbidden *métier* is set to 0.

2/ To avoid an unrealistic discarding peak the month the TAC is reached, the effort is altered when total landings/catches are close to the TAL/TAC value (TAL: hake and sole before 2016, Norway lobster; TAC: hake and sole from 2016). Details are provided in appendix A.3.

3/ To avoid an unrealistic early fishery closure, the catch is spread all over the whole year, using an expected catch for the year to come. Details are provided in appendix A.3.

2/ and 3/ are based on nominal effort times the inter-annual variation factor for the time step. Hence target factors *per se* are not included in these calculations.

2.5 Calibration

Accessibilities and targeting factors were calibrated on catch observations and fishing mortalities estimates, following a procedure exposed below.

Hake accessibility

$$
\begin{aligned}
FO &= \sum_{\substack{p\in P\\ s\in S\\ Smet\in SMET}} (\omega^{LFD}_{Smet,p,s} * \alpha * FO_1 + \omega^{weight}_{Smet,p,s} * (\beta * FO_2 + \gamma * FO_3)) \\
FO_1 &= (\frac{C^{obs}_{Smet,l,s,p}}{\sum\limits_{l\in L} C^{obs}_{Smet,l,s,p}} - \frac{C^{sim}_{Smet,l,s,p}}{\sum\limits_{l\in L} C^{sim}_{Smet,l,s,p}})^2 \\
FO_2 &= (\frac{\sum\limits_{l\in L} C^{obs}_{Smet,l,s,p} - \sum\limits_{l\in L} C^{sim}_{Smet,l,s,p}}{\sum\limits_{l\in L} C^{obs}_{Smet,l,s,p}})^2 \\
FO_3 &= (\frac{\sum\limits_{\substack{l\in L\\ s\in year}} C^{obs}_{Smet,l,s,p} - \sum\limits_{\substack{l\in L\\ s\in year}} C^{sim}_{Smet,l,s,p}}{\sum\limits_{\substack{l\in L,\\ s\in year}} C^{obs}_{Smet,l,s,p}})^2
\end{aligned}
\tag{14}
$$

, with FO_1, FO_2 and $FO_3$the 3 objective function components (first component focusing on length composition for each season and fleets ; the second focusing on catch in weight for each season and fleet; the third focusing on annual catch in weight); $\omega^{LFD}_{.,.,.}$ weightings on catch length composition observations, $\omega^{weight}_{.,.,.}$ weightings on weight observations. See Vigier et al. (2018); Vigier (2018) for the rationale behind the weightings derivation; $C^{sim}_{.,.,.,.}$ catch simulated in ISIS-Fish; $C^{obs}_{.,.,.,.}$ the observed catch. α, β et γ weightings on objective function components, respectively10, 1 and 35 (empirical), allowing the balance the influence of each objective function component, and favouring the third component, to improve the ability to reprocude annual catch in weight.

The calibration procedure consists in computing accessibility values minimizing the objective funtion using an iterative algorithm. At the beginning of the initial iteration, accessibilties values were set as inVigier et al. (2018). A batch of simulations was run, each using a multiplicator value applied to all hake accessibilities, varying between 0.5 and 2.5, with a 0.05 step. The objective function was derived then computed for all this values combinations and the optimal multiplicator value was identified for each parameter (quarter).

A new accessibility value was deduced, Next iteration is performed by multiplying the accessibility value of each quarter by their respective optimal multiplicator value.

Next iterations were initiated similarly as what was described in last paragraph. We stopped the estimation procedure when after an iteration, accessibilities values were not changed. Final values are provided in table S17.

Sole accessibility

$$OF = \sum_{a\in A y\in Y} (\frac{F^{obs}_{a,y}}{F^{sim}_{a,y}} - 1)^2 \tag{15}$$

, with A the set of age classes, Y the set of years, $F^{obs}_{.}$ the "observed" working group estimate, $F^{sim}_{.}$ the ISIS-Fish estimate.

At the beginning of the initial iteration, The calibration algorithm starts with accessibilties values set with values in the original database (V0). A single simulation was run, and the objective function was then computed. If it was deemed too high (*i.e.* $\frac{F^{obs}_{a,y}}{F^{sim}_{a,y}}$ too far from 1 for at least one age class), all accessibilties values were multiplitied The following tested values of sole accessibility is derived by multiplying the current accessibility values by $\frac{F^{obs}_{a,y}}{F^{sim}_{a,y}}$ ratio. Next iterations were initiated similarly as what was described in last paragraph.We The procedure was stopped the estimation procedure after 3 iterations, as all $\frac{F^{obs}_{a,y}}{F^{sim}_{a,y}}$ ratios were close to 1 (subjective). Final values are provided in table S18.

Norway lobster accessibility

$$OF_q = \sum_{s \in S l \in L} (\frac{C^{obs}_{s,l,q}}{C^{sim}_{s,l,q}} - 1)^2 \qquad (16)$$

, with q a quarter, L the set of length classes, S the set of sexes, $C^{obs}_{.,.,.}$ the observed catch in numbers, $C^{sim}_{.,.,.}$ the simulated catch in numbers

One estimation procedure was run for each quarter, in the order of quarters, as the catch done in the first quarter can influence the catch done in further quarters.

At the beginning of the initial iteration, accessibilties values were set as in the initial database (V0). A single simulation was run, and the objective function was then derived. If it was deemed to high (*i.e.* $\frac{C^{obs}_{s,l,q}}{C^{sim}_{s,l,q}}$ to far from 1 for at least sex*length class for quarter q), all accessibilties values were multiplitied by their respective $\frac{C^{obs}_{s,l,q}}{C^{sim}_{s,l,q}}$ ratio for quarter q. The estimation procedure was stopped within a quarter when all $\frac{C^{obs}_{s,l,q}}{C^{sim}_{s,l,q}}$ ratios were deemed close to 1 (subjective), which required 3 iterations for each quarter. Final values are provided in table S19.

Target factors $Tarf_{met,sp,q,y}$ drive how the effort is distributed between population sp, *métier met* and season*year (q,y) combinations. They were split in 3 components: a fixed component $Tarf_{met,sp,q}$ for each *métier met*, population sp and quarter q, derived from the effort dataset analysis, another fixed component $Tarf_{gmet,sp,q,y}$, for each group of *métiers gmet* sharing the same gear (longliners, gillnetters, roundfish trawler (coastal), roundfish trawler (not coastal), Norway lobster trawler), population sp, quarter q and year y, driving inter-annual variations of fishing effort, derived from catch observations (no quarter dimension for sole and Norway lobster for that component), and finally an estimated component, $Tarf^{est}_{gmet,sp,q}$, allowing to tune the model's dynamics to observed catch. This section focuses on the estimation of teh latter. The 3 components were combined as follows:

$$Tarf_{met,sp,q,y} = Tarf_{met,sp} * Tarf_{gmet,sp,q,y} * Tarf^{est}_{gmet,sp,q} \qquad (17)$$

As for accessibilities, target factors estimated components were estimated jointly within a population, but not between populations.

Hake target factors 20 parameters were defined, for each combination of the 5 five group of *métiers* (longliners, gillnetters, roundfish trawler (coastal), whitefish trawler (not coastal)) and 4 quarters. We used the same data and objective function as for hake's accessibilties estimation. At the beginning of the first iteration, each estimated component was set equal to the ratio of observed catch over simulated catch $\frac{C^{obs}_{gmet,sp,q}}{C^{sim}_{gmet,sp,q}}$. A set of 21 simulations was then launched, each of them with values of estimated components of the target factor varying between 0.5 times and 1.5 times the latter ratio, with a step of 0.05. After the runs, objective functions were derived, and an optimal ratio value (minimizing the objective function) was identified. If theses values were 1 for all target factors, the procedure was stopped. If not, a new iteration of this procedure was done, by defining the estimated components of target factors being equal to $\frac{C^{obs}_{gmet,sp,q}}{C^{sim}_{gmet,sp,q}}$ at the end of this iteration. Estimated values are provided in table S28.

Sole target factors : 1 estimated component per group of *métiers* (gillnetters, Norway lobster trawlers and whitefish trawlers) and quarter. We were provided with sole catch in weight on 2010-2012 for each *métier* and quarter. Just after hake's target factors estimation, a simulation was run, and for each group of *métiers* and quarter combination, the average of yearly ratios of observed over simulated catch of sole $\frac{C^{obs}_{gmet,sp,q}}{C^{sim}_{gmet,sp,q}}$ was derived. These ratios were used as multiplicative of target factors for sole ($Tarf^{est}_{gmet,sp,q}$). After a simulation using these latter target factors, all the latter ratios were close to 1, showing the catch distribution between *métiers* and quarters was more realistic. Hence no further iteration has been done. Estimated values are provided in table S29.

Norway lobster target factors : 1 estimated component per group of *métiers* (Norway lobster trawlers and whitefish trawlers). We were provided with monthly Norway lobster landings data per length and sex class for 2010. Just after sole's target factors estimation, a simulation was run, and for each group of *métiers*, the average of monthly ratios of observed over simulated catch of Norway lobster $\frac{C^{obs}_{gmet,sp,m}}{C^{sim}_{gmet,sp,m}}$ was derived. The average of these ratios for each group of *métiers* were used as multiplicative of target factors for Norway lobster ($Tarf^{est}_{gmet,sp,q}$). After a simulation using these latter target factors, all the latter ratios were close to 1, showing the landings distribution between *métiers* was more realistic. Hence no further iteration has been done. Estimated values are provided in table S30.

A Additional information about ISIS-Fish parametrisation

A.1 Biology

January (except first time step)

From \ To	BoB reproduction	BoB presence	BoB recruitment	BoB recruitment interim	Celtic Sea	northern area
Bay of Biscay reproduction	1	0	0	0	0	0
Bay of Biscay presence	M_l	1-M_l	0	0	0	0
Bay of Biscay recruitment	0	0	1-A	A	0	0
Bay of Biscay recruitment interim	M_l	0	0	1-M_l	0	0
Celtic Sea	0	0	0	0	1	0
northern area	0	0	0	0	0	1

April

Bay of Biscay reproduction	1-0.6M_l	0.6M_l	0	0	0	0
Bay of Biscay presence	0	1	0	0	0	0
Bay of Biscay recruitment	0	0	1-A	A	0	0
Bay of Biscay recruitment interim	0	0	0	1	0	0
Celtic Sea	0	0	0	0	1	0
northern area	0	0	0	0	0	1

July

Bay of Biscay reproduction	1-M_l	M_l	0	0	0	0
Bay of Biscay presence	0	1	0	0	0	0
Bay of Biscay recruitment	0	0	1-A	A	0	0
Bay of Biscay recruitment interim	0	0	0	1	0	0
Celtic Sea	0	0	0	0	1	0
northern area	0	0	0	0	0	1

October

From \ To	BoB reproduction	BoB presence	BoB recruitment	BoB recruitment interim	Celtic Sea	northern area
Bay of Biscay reproduction	1	0	0	0	0	0
Bay of Biscay presence	0	1	0	0	0	0
Bay of Biscay recruitment	0	0	1-A	A	0	0
Bay of Biscay recruitment interim	0	0	0	1	0	0
Celtic Sea	0	0	0	0	1	0
northern area	0	0	0	0	$0.3698M_l$	1-$0.3698M_l$

Other time steps (except first time step)

Bay of Biscay reproduction	1	0	0	0	0	0
Bay of Biscay presence	0	1	0	0	0	0
Bay of Biscay recruitment	0	0	1-A	A	0	0
Bay of Biscay recruitment interim	0	0	0	1	0	0
Celtic Sea	0	0	0	0	1	0
northern area	0	0	0	0	0	1

First time step

Bay of Biscay reproduction	1	0	0	0	0	0
Bay of Biscay presence	0	1	0	0	0	0
Bay of Biscay recruitment	0	0	1	0	0	0
Bay of Biscay recruitment interim	0	0	0	1	0	0
Celtic Sea	0	0	0	0	1	0
northern area	0	0	0	0	0	1

Table S11: Matrix of migrations modelled in ISIS-Fish. A=1 if $l > 20$cm, A=0 otherwise. Movement from recruitment to recruitment interim happens before all other movements

Initial abundance

Bin number	Celtic Sea	Northern area	BoB recruitment	BoB presence	BoB spawning	BoB recruitment interim
0	0	0	0	0	0	0
1	686.963505413608	820.403175147037	1473.88108452608	0	0	0
2	4299.01764353279	5143.45953005428	9252.42638753675	0	0	0
3	18803.6263704317	22516.9669651367	40556.0292972695	0	0	0
4	67401.473103636	80741.093826629	145534.091131117	0	0	0
5	200343.012277552	240032.503946362	432791.427010025	0	0	0
6	495055.866217123	593178.562145975	1069499.18200658	0	0	0
7	1020597.00082046	1222929.09344847	2204123.76344918	0	0	0
8	1765567.11330918	2115612.71105415	3810002.81997532	0	0	0
9	2585949.34855167	3098593.56040527	5572333.76685415	0	0	0
10	3250975.15894442	3895176.17007348	6988116.02759652	0	0	0
11	3582269.48299457	4291266.59733648	7668714.29494604	0	0	0
12	3567353.56597557	4271279.12294742	7586889.11802565	0	0	0
13	3340277.02978929	3994816.41566441	7035221.51992591	0	0	0
14	3061168.56323078	3652276.21043729	6364323.26530271	0	0	0
15	2794601.59262899	3319583.2862153	5721509.617112	0	0	0
16	2578630.01798934	3039419.81958298	5184732.42885618	0	0	0
17	2397632.13872207	2789779.38022965	4714343.02561483	0	0	0
18	2216287.02987263	2525189.22751526	4229566.64644247	0	0	0
19	2039400.43209399	2247872.20714494	0	0	42238.4018526145	3689808.37116926
20	1893509.49902698	1985980.29697018	0	0	45073.2739939744	3223714.27876227
21	1807175.7931907	1770346.80043179	0	0	48553.2905709868	2843133.21967856
22	1799344.98151629	1621025.38206088	0	0	53814.3039138023	2579986.13490605
23	1876830.49281737	1544827.80462448	0	0	62228.2151298419	2442576.04213133
24	2037011.63351687	1539091.59676212	0	0	75370.2385233111	2422153.94342326
25	2271409.09005875	1596382.84441154	0	0	95065.3731393845	2501297.29246454
26	2567866.3048584	1707319.23428027	0	0	123441.957635022	2659174.63738542
27	2911214.95153137	1861311.82979442	0	0	162910.571916476	2873256.7218368
28	3283376.40667329	2046332.74134318	0	0	216020.743425086	3119332.88006462
29	3663789.67464337	2248801.60168893	0	0	285191.011937683	3371655.5812005
30	4030544.44208227	2454091.67526088	0	0	372344.387273098	3604070.56528668
31	4362069.7837493	2647592.93927233	0	0	478501.540610397	3792040.07095587
32	4639007.97143426	2815979.395297	0	0	603389.195057541	3914967.94166035
33	4845892.248146	2948365.89838994	0	0	745127.191864088	3958240.67342444
34	4972328.69410926	3037106.75679293	0	0	900055.184186455	3914551.63595943
35	5013616.80009389	3078182.20473767	0	0	1062766.68716167	3784355.48116573
36	4970751.65577035	3071184.32817571	0	0	1226392.11600991	3575398.43394869
37	4849879.68680869	3018952.02718619	0	0	1383147.4530155	3301449.66762717
38	4661322.55295136	2926952.52612251	0	0	1525111.43409025	2980429.68262777
39	8497856.15162561	5426502.36562481	0	0	3366097.39734594	4873215.71846585
40	7325701.94206166	4799866.05542899	0	0	3630121.48110136	3522835.17757331
41	6092909.02530043	4128094.22594219	0	0	3646566.4032161	2372124.60980457
42	4967764.84917387	3504953.15212007	0	0	3470854.28566742	1513463.53419132
43	4029480.47378784	2974203.67097859	0	0	3183106.85663837	930398.508160448
44	3286602.47967446	2539450.34458917	0	0	2848609.06115946	558126.760207029
45	2707520.38661035	2182360.20766286	0	0	2503295.09872097	328771.566378988
46	2249045.50304923	1879664.89014934	0	0	2161686.42376543	190308.025784625
47	1874422.47373185	1613504.11951837	0	0	1830801.61275273	108040.827671166
48	1559685.89861314	1374428.24813917	0	0	1518343.32905427	60061.8643994374
49	1292028.74861472	1159513.3599367	0	0	1233572.63378812	32709.6394392912
50	1065075.65715264	968823.879826939	0	0	984536.976427469	17499.4850706001
51	874635.682828918	802526.149312314	0	0	775424.507545815	9238.78728718262
52	716409.862739799	659566.644958148	0	0	605788.148917453	4838.13993399307
53	585529.664992637	537695.924291744	0	0	471516.779908318	2524.27701186793
54	477080.519100549	434147.939777138	0	0	366612.350276891	1315.61603489883
55	386770.167769543	346333.425798456	0	0	284854.550222666	685.215905343013
56	311294.698531661	272201.71095637	0	0	220860.330211309	356.126423754206
57	248307.149127122	210249.553435215	0	0	170470.708550704	184.254649150628
58	196144.989167441	159313.826549377	0	0	130672.81178495	94.6751783846533
59	153507.70578349	118328.829556369	0	0	99309.565728061	48.2307689328954
60	119215.144350031	86164.7328052579	0	0	74773.9616666564	24.3425292944647
61	92094.276984655	61582.0544698776	0	0	55785.8373259796	12.173668563945
62	70971.4317506614	43278.4557777114	0	0	41267.9586408275	6.03660433710039
63	54720.8253511442	29978.3009230452	0	0	30296.434595038	2.97066345812836
64	42323.0055873732	20519.6182552369	0	0	22087.3753060591	1.45173801714275
65	32906.1671575701	13913.0216431132	0	0	15994.0982226468	0.704670523538202
66	25759.484108479	9363.8702233789	0	0	11499.9043045055	0.33962744673557
67	20325.1291841014	6264.76754316594	0	0	8203.47338340053	0.162400938376241
68	16176.2875184831	4169.82340984203	0	0	5799.46825577609	0.076959306202477
69	44395.49557175	6894.14167602662	0	0	10702.6960832025	0.042777242251272
70	18044.8884661231	946.023395902999	0	0	1642.14886380748	0.000888266227693
71	9005.18241326109	97.9378377761283	0	0	187.977793120593	1.37609434132964E-05

Table S13: Initial time step hake abundance in numbers by zone (column) and size bin (row)

Age	2	3	4	5	6	7	≥ 8
Abundance (thousands)	24110	28001	8655	3873	2027	1305	1375

Table S14: Initial time step sole abundance by age bin (ICES, 2017)

Bin number	21E7	22E6	22E7	23E5	23E6	24E5	24E6	21E6	20E7	20E8
0	58665802.8528978	103697427.541198	13849598.2851683	64723062.3015429	98473950.81997	82703000.8761373	19583004.2579025	13662228.4678046	7346369.29525232	15335094.4156574
1	25056509.5954154	44289781.4715165	5915244.92036893	27643600.7476061	42058803.8282879	35322938.3090293	8364016.34740359	5835218.16886538	3137677.55296069	6549709.05854637
2	0	0	0	0	0	0	0	0	0	0
3	0	0	0	0	0	0	0	0	0	0
4	77499.0087621374	136986.923468472	18295.6694813446	85500.8016339092	130086.57865938	109252.755061175	25869.6437237489	18048.1492155087	9704.73957051684	20258.0474261511
5	439161.049652112	776259.232988009	103675.460394286	484504.542592152	737157.279069817	619098.945346658	146594.64776791	102272.845554549	54993.5242329287	114795.602081523
6	2118306.23949842	3744309.24147157	500081.632490087	2337021.91132685	3555699.81668971	2986241.97167212	707103.595115803	493316.078557237	265262.881594127	553719.962981465
7	2686632.3037541	4748880.0135737	634249.875353281	2964027.78997552	4509668.06019183	3787428.84212073	896814.315756628	625669.172804301	336430.971777917	702278.977439906
8	6006173.17906566	10616486.5688066	1417914.38480421	6626312.12662798	10081709.8461019	8467088.51724108	2004897.38859054	1398731.56420193	752117.316715057	1569998.67552672
9	6199920.70097101	10958953.8774778	1463653.55850758	6840064.13071276	10406926.2927504	8740220.40489403	2069571.49789992	1443851.9372407	776379.16564135	1620643.7940921
10	4998686.06515786	8835656.56371645	1180070.68154673	5514801.70538714	8390584.32352997	7046802.70144578	1668592.0201818	1164105.62440031	625955.702298335	1306644.05898675
11	4482026.00674363	7922410.40726	1058099.5516711	4944796.3611611	7523340.46580081	6318451.00103797	1496127.72869015	1043784.62963025	561257.438494892	1171590.40947908
12	2027890.72927593	3584491.16409169	478736.684761852	2237270.97608729	3403932.1415871	2858780.42410075	676922.344104763	472259.904472477	253940.685428524	530085.574317622
13	2144139.24241914	3789971.5492944	506180.188983869	2365522.17853816	3599062.00957617	3022659.55669251	715726.809690386	499332.12829574	268497.794784299	560472.645456848
14	1304566.64749598	2305946.54505262	307977.102935969	1439263.49417081	2189790.74076623	1839088.04352978	435472.33601644	303810.511794397	163363.1161037	341010.465006878
15	852489.09638351	1506856.15815319	201252.364294791	940508.817973	1430952.36525317	1201780.30567292	284566.080961237	198529.641370595	106752.135275685	222838.521687662
16	632908.571557454	1118726.54165919	149414.634097648	698256.546676924	1062373.72571826	892230.832999594	211268.757077282	147393.21859332	79255.3731592207	165440.720646901
17	607075.568636745	1073064.23383637	143316.077603867	669756.279465624	1019011.53283181	855813.247979206	202645.5425027	141377.168854818	76020.4599690488	158688.038171518
18	529576.559874605	936077.310367891	125020.408122521	584255.477831712	888924.954172425	746560.492918027	176775.89877895	123329.019639309	66315.7203985316	138429.990745366
19	400411.545271042	707765.77125377	94527.6256536136	441754.141775196	672113.989740125	564472.567816068	133659.825906035	93248.7709467944	50141.1544476701	104666.578368447
20	322912.536508906	570778.847785301	76231.9561722694	356253.340141289	542027.411080749	455219.812754896	107790.182182287	75200.6217312862	40436.4148771535	84408.5309422965
21	38749.5043810689	68493.4617342364	9147.83474067236	42750.4008169548	65043.2893296901	54626.3775305877	12934.8218618745	9024.07460775438	4852.36978525844	10129.0237130756
22	25833.0029207125	45662.3078228241	6098.55649378155	28500.2672113031	43362.1928864599	36417.5850203917	8623.21457458297	6016.04973850289	3234.91319017228	6752.68247538372
23	0	0	0	0	0	0	0	0	0	0
24	64582.5073017811	114155.76955706	15246.3912344539	71250.6680282576	108405.482216149	91043.962550979	21558.0364364574	15040.1243462572	8087.28297543069	16881.7061884593
25	0	0	0	0	0	0	0	0	0	0
26	0	0	0	0	0	0	0	0	0	0
27	0	0	0	0	0	0	0	0	0	0
28	0	0	0	0	0	0	0	0	0	0
29	6625.99750239055	11712.085448062	1564.24013963878	7310.13347302905	11122.1209026959	9340.87407990567	2211.7985434807	1543.07769266795	829.734227349385	1732.01920634842
30	0	0	0	0	0	0	0	0	0	0
31	0	0	0	0	0	0	0	0	0	0
32	0	0	0	0	0	0	0	0	0	0
33	0	0	0	0	0	0	0	0	0	0
34	0	0	0	0	0	0	0	0	0	0
35	25056509.5954154	44289781.4715165	5915244.92036893	27643600.7476061	42058803.8282879	35322938.3090293	8364016.34740359	5835218.16886538	3137677.55296069	6549709.05854637
36	0	0	0	0	0	0	0	0	0	0
37	77499.0087621374	136986.923468472	18295.6694813446	85500.8016339092	130086.57865938	109252.755061175	25869.6437237489	18048.1492155087	9704.73957051684	20258.0474261511
38	180831.020444987	319636.154759769	42689.8954564709	199501.870479122	303535.350205219	254923.095142742	60362.5020220808	42112.3481695202	22644.392331206	47268.777327686
39	284163.032127837	502285.386051065	67084.1214315971	313502.939324334	476984.121751059	400593.435224308	94855.3603204126	66176.5471235318	35584.0450918951	74279.5072292209
40	1356232.65333741	2397271.16069826	320174.215923531	1496264.02859341	2276515.12653914	1911923.21357056	452718.765165606	315842.611271402	169832.942484045	354515.829957645
41	4301194.98629864	7602774.25250024	1015409.65621463	4745294.49068198	7219805.11559559	6063527.90589523	1435765.22666807	1001672.28146074	538613.046163687	1124321.63215139
42	7284906.82364093	12876770.8060364	1719792.9312464	8037075.35358748	12228138.3939817	10269758.9757505	2431746.5100324	1696526.02625782	912245.519628585	1904256.45805821
43	11172773.7632081	19748948.1333714	2637625.68356052	12326365.5688886	18754148.4233939	15750605.5213194	3729540.30350713	2601941.5119025	1399099.95474951	2920535.17060346
44	6845745.77398881	12100511.5730484	1616117.47085211	7552570.81099532	11490981.1149119	9650660.0304038	2285151.86226449	1594253.18070327	857251.995395655	1789460.85597669
45	3991198.95125009	7054826.55862634	942226.978289253	4403291.28414634	6699458.80095807	5626516.88565053	1332286.65177307	929479.6845987	499794.087881619	1043289.44244679
46	1640395.68546524	2899556.54674933	387258.337355129	1809766.96791775	2753499.24829021	2312516.64879487	547574.125486019	382019.158394934	205416.98757594	428795.337186867
47	1304566.64749598	2305946.54505262	307977.102935969	1439263.49417081	2189790.74076623	1839088.04352978	435472.33601644	303810.511794397	163363.1161037	341010.465006878
48	516660.058414248	913246.156456479	121971.129875631	570005.34422606	867243.857729195	728351.700407831	172464.291491659	120320.994770057	64698.2638034454	135053.649507674
49	258330.029207125	456623.078228241	60985.5649378155	285002.672113031	433621.928864599	364175.850203917	86232.1457458296	60160.4973850289	32349.1319017228	67526.8247538372
50	180831.020444987	319636.154759769	42689.8954564709	199501.870479122	303535.350205219	254923.095142742	60362.5020220808	42112.3481695202	22644.392331206	47268.777327686
51	90415.5102224937	159818.077379884	21344.9477282354	99750.9352395608	151767.67510261	127461.547571371	30181.2510110404	21056.1740847601	11322.196165603	23634.388663843
52	25833.0029207125	45662.3078228241	6098.55649378155	28500.2672113031	43362.1928864599	36417.5850203917	8623.21457458297	6016.04973850289	3234.91319017228	6752.68247538372
53	0	0	0	0	0	0	0	0	0	0
54	25833.0029207125	45662.3078228241	6098.55649378155	28500.2672113031	43362.1928864599	36417.5850203917	8623.21457458297	6016.04973850289	3234.91319017228	6752.68247538372
55	25833.0029207125	45662.3078228241	6098.55649378155	28500.2672113031	43362.1928864599	36417.5850203917	8623.21457458297	6016.04973850289	3234.91319017228	6752.68247538372
56	0	0	0	0	0	0	0	0	0	0
57	0	0	0	0	0	0	0	0	0	0

Table S16: Initial time step Norway lobster abundance in numbers by zone (column) and size*sex bin (row). 0 is the recruitment bin, male bins go from 1 to 33, female bins from 35 to 57. 34 bin is not used.

Accessibilities

Quarter 1	Quarter 2	Quarter 3	Quarter 4
3.079678E-6	1.516711E-6	1.303504E-6	1.435228E-6

Table S17: Estimated accessibilities for hake, for each quarter (column). Accessibilites are constant across length groups for hake.

Age group	Accessibility
2	2.89499380291302E-7
3	1.00932912677375E-6
4	1.6945138111703E-6
5	1.44361782815352E-6
6	1.05318878269456E-6
7	6.46001925336324E-7
8+	6.45937924572751E-7

Table S18: Estimated accessibilities for sole, for each age group (rows).

Length-sex group	January	February-March	April	May-June	July-August	September	October	November-December
0	0.0	0.0	0.0	0.0	0.0	0.0	0.0	0.0
1	3.18846915088803E-8	3.18846915088803E-8	0.0	0.0	0.0	0.0	0.0	0.0
2	0.0	0.0	0.0	0.0	0.0	0.0	0.0	0.0
3	3.23945063622126E-5	3.23945063622126E-5	0.0	0.0	0.0	0.0	8.11356384207909E-7	8.11356384207909E-7
4	9.31317087307513E-4	9.31317087307513E-4	4.55016739913101E-6	4.55016739913101E-6	2.33283327746362E-6	2.33283327746362E-6	2.40369544540296E-7	2.40369544540296E-7
5	4.95550143584557E-5	4.95550143584557E-5	1.36620705394768E-5	1.36620705394768E-5	1.42470572448755E-5	1.42470572448755E-5	4.77148621733561E-6	4.77148621733561E-6
6	1.09987301775807E-5	1.09987301775807E-5	2.31443394701062E-5	2.31443394701062E-5	2.44100816449602E-5	2.44100816449602E-5	9.03395965647114E-6	9.03395965647114E-6
7	1.61758526495058E-5	1.61758526495058E-5	2.95969706201094E-5	2.95969706201094E-5	3.71931190047177E-5	3.71931190047177E-5	1.77308605546832E-5	1.77308605546832E-5
8	5.43502015320795E-6	5.43502015320795E-6	2.24487285655068E-5	2.24487285655068E-5	2.89356876071471E-5	2.89356876071471E-5	1.80136396768007E-5	1.80136396768007E-5
9	4.64683086554677E-6	4.64683086554677E-6	1.58387051479566E-5	1.58387051479566E-5	2.73546310997068E-5	2.73546310997068E-5	2.67769455840013E-5	2.67769455840013E-5
10	5.65550601536275E-6	5.65550601536275E-6	1.37687247132791E-5	1.37687247132791E-5	2.15433223679305E-5	2.15433223679305E-5	2.94258724458217E-5	2.94258724458217E-5
11	4.96528651967682E-6	4.96528651967682E-6	1.42344896795448E-5	1.42344896795448E-5	2.13362701240694E-5	2.13362701240694E-5	2.63798772436162E-5	2.63798772436162E-5
12	8.66724955534049E-6	8.66724955534049E-6	9.61392146546276E-6	9.61392146546276E-6	1.1667984790533E-5	1.1667984790533E-5	1.66221901195554E-5	1.66221901195554E-5
13	4.86129294805153E-6	4.86129294805153E-6	8.90724091464622E-6	8.90724091464622E-6	1.03413976461096E-5	1.03413976461096E-5	1.11929656188697E-5	1.11929656188697E-5
14	3.97896646337541E-6	3.97896646337541E-6	5.30597967867694E-6	5.30597967867694E-6	6.03980124621398E-6	6.03980124621398E-6	7.55762696050965E-6	7.55762696050965E-6
15	3.56626886285801E-6	3.56626886285801E-6	3.92969137750258E-6	3.92969137750258E-6	4.5637305300016E-6	4.5637305300016E-6	4.89307871470057E-6	4.89307871470057E-6
16	3.85661770750293E-6	3.85661770750293E-6	3.21537516718379E-6	3.21537516718379E-6	3.26831780801961E-6	3.26831780801961E-6	3.50074017008579E-6	3.50074017008579E-6
17	1.70122335755978E-6	1.70122335755978E-6	2.45599723053603E-6	2.45599723053603E-6	2.76280209221792E-6	2.76280209221792E-6	2.38620150628067E-6	2.38620150628067E-6
18	1.56262842177495E-6	1.56262842177495E-6	1.98542306243037E-6	1.98542306243037E-6	2.84633312631908E-6	2.84633312631908E-6	2.15925963248582E-6	2.15925963248582E-6
19	1.62382639277463E-6	1.62382639277463E-6	1.60288632994993E-6	1.60288632994993E-6	2.24544709381552E-6	2.24544709381552E-6	1.21826370563599E-6	1.21826370563599E-6
20	1.68083604362574E-6	1.68083604362574E-6	1.47681170936384E-6	1.47681170936384E-6	2.31017543686603E-6	2.31017543686603E-6	1.05859271898768E-6	1.05859271898768E-6
21	1.55782295919582E-5	1.55782295919582E-5	1.64669884717797E-6	1.64669884717797E-6	2.01735601579392E-6	2.01735601579392E-6	1.06409234222854E-6	1.06409234222854E-6
22	1.30466718137872E-5	1.30466718137872E-5	4.47680628913875E-6	4.47680628913875E-6	2.88521813581566E-6	2.88521813581566E-6	8.61413454733327E-7	8.61413454733327E-7
23	3.23945063622126E-5	3.23945063622126E-5	6.44131407915379E-6	6.44131407915379E-6	5.64796563057958E-6	5.64796563057958E-6	2.09383562586113E-6	2.09383562586113E-6
24	3.03090666408513E-6	3.03090666408513E-6	7.1136219048505E-6	7.1136219048505E-6	6.26066234437771E-6	6.26066234437771E-6	1.99360178215248E-6	1.99360178215248E-6
25	3.23945063622126E-5	3.23945063622126E-5	5.68988676772377E-6	5.68988676772377E-6	6.55983914501277E-6	6.55983914501277E-6	2.84061959149694E-6	2.84061959149694E-6
26	3.23945063622126E-5	3.23945063622126E-5	1.66966573414544E-5	1.66966573414544E-5	2.98022450143279E-5	2.98022450143279E-5	4.40404583362494E-6	4.40404583362494E-6
27	3.23945063622126E-5	3.23945063622126E-5	1.28854352536265E-5	1.28854352536265E-5	2.45835106283426E-5	2.45835106283426E-5	2.92911472728058E-6	2.92911472728058E-6
28	3.23945063622126E-5	3.23945063622126E-5	7.20823895413871E-5	7.20823895413871E-5	5.36543102634057E-4	5.36543102634057E-4	2.37527466495165E-5	2.37527466495165E-5
29	5.45391226149533E-6	5.45391226149533E-6	1.32300771407626E-6	1.32300771407626E-6	2.61629815026469E-6	2.61629815026469E-6	2.37154364258359E-6	2.37154364258359E-6
30	3.23945063622126E-5	3.23945063622126E-5	2.34011228971957E-6	2.34011228971957E-6	5.3354103414247E-6	5.3354103414247E-6	0.0	0.0
31	0.0	0.0	0.0	0.0	0.00530424024193342	0.00530424024193342	1.15638674038637E-5	1.15638674038637E-5
32	0.0	0.0	0.0	0.0	0.0	0.0	0.0	0.0
33	0.0	0.0	0.0	0.0	0.0	0.0	0.0	0.0
34	0.0	0.0	0.0	0.0	0.0	0.0	0.0	0.0
35	0.0	0.0	0.0	0.0	0.0	0.0	0.0	0.0
36	3.23945063622126E-5	3.23945063622126E-5	0.0	0.0	0.0	0.0	3.00573333643139E-6	3.00573333643139E-6
37	1.53697575442122E-4	1.53697575442122E-4	0.0	0.0	5.84927640007637E-7	5.84927640007637E-7	1.13881753832024E-6	1.13881753832024E-6
38	1.87597783044398E-4	1.87597783044398E-4	2.14204964447498E-6	2.14204964447498E-6	3.85174863110581E-6	3.85174863110581E-6	2.27586582199014E-6	2.27586582199014E-6
39	6.09278631516737E-5	6.09278631516737E-5	8.48348551806047E-6	8.48348551806047E-6	2.07458976209801E-5	2.07458976209801E-5	5.95125023275123E-6	5.95125023275123E-6
40	2.60411820628756E-5	2.60411820628756E-5	2.38285261835481E-5	2.38285261835481E-5	3.75018116458704E-5	3.75018116458704E-5	1.50651251071956E-5	1.50651251071956E-5
41	9.22733205876687E-6	9.22733205876687E-6	1.85212690663184E-5	1.85212690663184E-5	2.723566891403E-5	2.723566891403E-5	1.1869049916607E-5	1.1869049916607E-5
42	4.74498125224341E-6	4.74498125224341E-6	1.57288612765162E-5	1.57288612765162E-5	1.60717691100172E-5	1.60717691100172E-5	6.16346724220225E-6	6.16346724220225E-6
43	2.65553340840955E-6	2.65553340840955E-6	1.02144718384041E-5	1.02144718384041E-5	8.58354265424235E-6	8.58354265424235E-6	1.64772546981725E-6	1.64772546981725E-6
44	3.03436439303652E-6	3.03436439303652E-6	8.32461306924601E-6	8.32461306924601E-6	5.23112002379585E-6	5.23112002379585E-6	7.67621941932616E-7	7.67621941932616E-7
45	2.09896097248297E-6	2.09896097248297E-6	8.53629909300745E-6	8.53629909300745E-6	4.41065019706335E-6	4.41065019706335E-6	5.88183580026362E-7	5.88183580026362E-7
46	1.99476013271777E-6	1.99476013271777E-6	7.78814609845942E-6	7.78814609845942E-6	3.92830744774213E-6	3.92830744774213E-6	5.15294858454565E-7	5.15294858454565E-7
47	5.44727997411463E-7	5.44727997411463E-7	6.15522993560403E-6	6.15522993560403E-6	3.31895014057977E-6	3.31895014057977E-6	3.51715299949725E-7	3.51715299949725E-7
48	9.65025858349912E-7	9.65025858349912E-7	4.03723624882389E-6	4.03723624882389E-6	2.91856870262172E-6	2.91856870262172E-6	2.86447755458256E-7	2.86447755458256E-7
49	1.04798201173862E-6	1.04798201173862E-6	4.20397252048312E-6	4.20397252048312E-6	3.77291425326462E-6	3.77291425326462E-6	7.6583677525899E-7	7.6583677525899E-7
50	5.73176352032734E-7	5.73176352032734E-7	4.4963390389232E-6	4.4963390389232E-6	3.3991823213466E-6	3.3991823213466E-6	8.62193978445789E-7	8.62193978445789E-7
51	1.44067933333024E-6	1.44067933333024E-6	2.85642953785505E-6	2.85642953785505E-6	4.8700488992478E-6	4.8700488992478E-6	5.41748870528773E-7	5.41748870528773E-7
52	2.86286000728438E-7	2.86286000728438E-7	5.37934543513673E-6	5.37934543513673E-6	8.48096765824588E-6	8.48096765824588E-6	1.80521087133569E-6	1.80521087133569E-6
53	3.23945063622126E-5	3.23945063622126E-5	1.60448813937538E-5	1.60448813937538E-5	3.14083577554604E-5	3.14083577554604E-5	1.41698636278211E-5	1.41698636278211E-5
54	4.77348332316162E-7	4.77348332316162E-7	5.8658875924446E-6	5.8658875924446E-6	8.67179644125098E-6	8.67179644125098E-6	5.42852306078785E-6	5.42852306078785E-6
55	1.90900759021613E-7	1.90900759021613E-7	3.30815868513166E-6	3.30815868513166E-6	4.02267655023739E-6	4.02267655023739E-6	2.94387819757337E-6	2.94387819757337E-6
56	0.0	0.0	1.32684227750366E-5	1.32684227750366E-5	6.10796048643364E-4	6.10796048643364E-4	5.77689689876829E-6	5.77689689876829E-6
57	3.23945063622126E-5	3.23945063622126E-5	6.1347839828047E-4	6.1347839828047E-4	6.10796048643364E-4	6.10796048643364E-4	0.0	0.0

Table S19: Estimated accessibilities for Norway lobster, for each season (column) and length-sex group (rows; 0 = mixed class; 1-33 male classes; 34 void class; 35-57 female classes; male and female classes are ordered by increasing length).

A.2 Exploitation

Formulæ for selectivity and retention functions From Methot and Wetzel (2013):

Logistic:

$$f(l) = \frac{1}{1 + e^{-ln(19)*\frac{L'_l - p_1}{p_2}}} \tag{18}$$

Double normal:

$$\begin{aligned}
f(l) &= asc_l * (1 - j_{1,l}) + j_{1,l} * ((1 - j_{2,l}) + j_{2,l} * dsc_l) \\
j_{1,l} &= \frac{1}{1 + e^{-20*\frac{L'_l - \mathbf{p_1}}{1+|L'_l - \mathbf{p_1}|}}} \\
j_{2,l} &= \frac{1}{1 + e^{-20*\frac{L'_l - peak}{1+|L'_l - peak|}}} \\
peak &= \mathbf{p_1} + L_{width} + \frac{0.99L'_{max} - \mathbf{p_1} - L_{width}}{1 + e^{-\mathbf{p_2}}} \\
asc_l &= \begin{cases} \frac{1}{1+e^{-\mathbf{p_5}}} + (1 - \frac{1}{1+e^{-\mathbf{p_5}}}) * \frac{e^{\frac{-(L'_l - \mathbf{p_1})^2}{e^{\mathbf{p_3}}}} - T_1}{1 - T_1} & , \mathbf{p_5} \neq -999 \\ T_1 & , \mathbf{p_5} = -999 \end{cases} \\
dsc_l &= \begin{cases} 1 + (\frac{1}{1+e^{-\mathbf{p_6}}} - 1) * \frac{e^{\frac{-(L'_l - peak)^2}{e^{\mathbf{p_4}}}}}{T_2 - 1} & , \mathbf{p_6} \neq -999 \\ T_2 & , \mathbf{p_6} = -999 \end{cases} \\
T_1 &= e^{-\frac{(L_{min} - \mathbf{p_1})^2}{e^{\mathbf{p_3}}}} \\
T_2 &= e^{-\frac{(L_{max} - peak)^2}{e^{\mathbf{p_4}}}}
\end{aligned} \tag{19}$$

Métiers zones

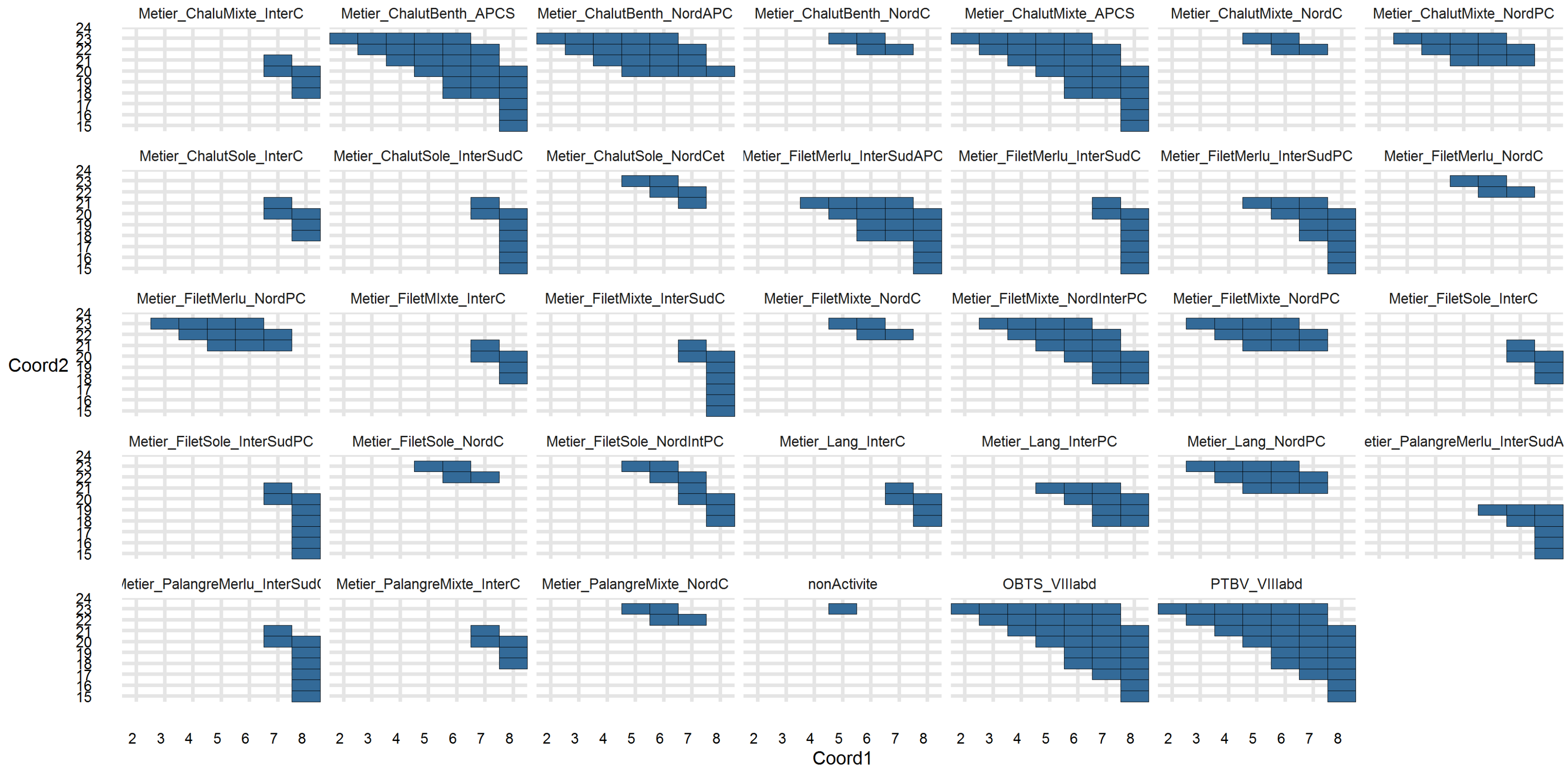

Figure S3: Bay of Biscay *métiers* zones

Strategy	Jan	Feb	Mar	Apr	May	Jun	Jul	Aug	Sep	Oct	Nov	Dec
SpainMainHakeON_20_29	8	8	8	8	8	8	8	8	8	8	8	8
SpainMainHakeON_30_39	8	8	8	8	8	8	8	8	8	8	8	8
SpainMainHakePA_30_39	8	8	8	8	8	8	8	8	8	8	8	8
SpainMainMegrimAnglerfishON_20_29	8	8	8	8	8	8	8	8	8	8	8	8
SpainMainMegrimAnglerfishON_30_39	8	8	8	8	8	8	8	8	8	8	8	8
SpainMainMegrimAnglerfishPA_30_39	8	8	8	8	8	8	8	8	8	8	8	8
STR_GdG_Nord_1218m_G	24	21	22	23	22	19	21	23	23	25	23	24
STR_GdG_Nord_1218m_O	21	20	19	19	20	19	21	20	21	22	21	24
STR_GdG_Nord_moins12m_G	25	21	22	21	22	20	20	21	21	23	22	25
STR_GdG_Nord_moins12m_G-L	17	15	20	14	13	12	14	12	13	16	16	17
STR_GdG_Nord_moins12m_O	22	18	18	15	16	14	15	14	14	17	17	22
STR_GdG_Nord_plus18m	20	16	18	18	18	18	18	19	19	21	22	23
STR_GdG_Sud_1218m_G	12	10	12	12	12	11	12	12	12	14	14	18
STR_GdG_Sud_1218m_O	12	12	12	11	14	12	12	10	11	12	12	15
STR_GdG_Sud_moins12m_G	14	11	15	12	14	12	14	14	14	18	18	18
STR_GdG_Sud_moins12m_G-L-O	17	14	14	10	13	11	14	14	15	17	16	19
STR_GdG_Sud_moins12m_L	16	17	17	13	12	12	16	15	15	17	16	19
STR_GdG_Sud_moins12m_O	15	12	17	16	16	14	15	14	14	17	18	17
STR_GdG_Sud_plus18m_G	11	9	12	17	12	13	14	12	13	15	13	18
STR_GdG_Sud_plus18m_O	15	14	15	14	16	16	17	15	16	15	15	17

Table S20: Number of inactive days per months depending on strategies

	January	February	March	April	May	June	July	August	September	October	November	December
nonActivite	0.0	0.0	0.0	0.0	0.0	0.0	0.0	0.0	0.0	0.0	0.0	0.0
PTBV˙VIIIabd	0.7	0.7	0.8	0.9	1.0	0.9	1.0	0.6	0.8	1.0	0.9	0.8
OBTS˙VIIIabd	0.0	0.0	0.0	0.0	0.0	0.0	0.0	0.0	0.0	0.0	0.0	0.0

Strategy SpainMainHakeON_20_29

	January	February	March	April	May	June	July	August	September	October	November	December
nonActivite	0.0	0.0	0.0	0.0	0.0	0.0	0.0	0.0	0.0	0.0	0.0	0.0
PTBV˙VIIIabd	0.6	0.7	0.8	0.8	0.9	0.9	0.9	0.6	0.9	1.0	1.0	0.9
OBTS˙VIIIabd	0.0	0.0	0.0	0.0	0.0	0.0	0.0	0.0	0.0	0.0	0.0	0.0

Strategy SpainMainHakeON_30_39

	January	February	March	April	May	June	July	August	September	October	November	December
nonActivite	0.0	0.0	0.0	0.0	0.0	0.0	0.0	0.0	0.0	0.0	0.0	0.0
PTBV˙VIIIabd	0.5	0.7	0.9	0.8	0.9	0.9	1.0	0.9	0.9	0.9	0.9	0.7
OBTS˙VIIIabd	0.0	0.0	0.0	0.0	0.0	0.0	0.0	0.0	0.0	0.0	0.0	0.0

Strategy SpainMainHakePA_30_39

	January	February	March	April	May	June	July	August	September	October	November	December
nonActivite	0.0	0.0	0.0	0.0	0.0	0.0	0.0	0.0	0.0	0.0	0.0	0.0
PTBV˙VIIIabd	0.0	0.0	0.0	0.0	0.0	0.0	0.0	0.0	0.0	0.0	0.0	0.0
OBTS˙VIIIabd	0.1	0.11	0.1	0.08	0.08	0.07	0.07	0.04	0.07	0.08	0.1	0.1

Strategy SpainMainMegrimAnglerfishON_20_29

	January	February	March	April	May	June	July	August	September	October	November	December
nonActivite	0.0	0.0	0.0	0.0	0.0	0.0	0.0	0.0	0.0	0.0	0.0	0.0
PTBV˙VIIIabd	0.0	0.0	0.0	0.0	0.0	0.0	0.0	0.0	0.0	0.0	0.0	0.0
OBTS˙VIIIabd	0.12	0.13	0.12	0.08	0.07	0.06	0.05	0.03	0.05	0.08	0.12	0.12

Strategy SpainMainMegrimAnglerfishON_30_39

	January	February	March	April	May	June	July	August	September	October	November	December
nonActivite	0.0	0.0	0.0	0.0	0.0	0.0	0.0	0.0	0.0	0.0	0.0	0.0
PTBV˙VIIIabd	0.0	0.0	0.0	0.0	0.0	0.0	0.0	0.0	0.0	0.0	0.0	0.0
OBTS˙VIIIabd	0.04	0.1	0.1	0.12	0.08	0.11	0.09	0.01	0.07	0.08	0.1	0.12

Strategy SpainMainMegrimAnglerfishPA_30_39

	January	February	March	April	May	June	July	August	September	October	November	December
Metier˙ChalutMixte˙APCS	0.0	0.0	0.0	0.0	0.0	0.0	0.0	0.0	0.0	0.0	0.0	0.0
Metier˙Lang˙NordPC	0.05	0.0	0.0	0.0	0.04	0.0	0.0	0.05	0.05	0.05	0.04	0.03
Metier˙ChalutSole˙NordCet	0.01	0.0	0.0	0.0	0.0	0.02	0.0	0.0	0.0	0.0	0.0	0.0
Metier˙ChalutBenth˙NordAPC	0.0	0.0	0.0	0.0	0.0	0.0	0.0	0.0	0.0	0.0	0.0	0.0
Metier˙FiletSole˙NordC	0.06	0.15	0.0	0.0	0.0	0.0	0.0	0.0	0.0	0.0	0.0	0.0
Metier˙FiletSole˙NordIntPC	0.62	0.71	0.68	0.51	0.34	0.37	0.37	0.46	0.48	0.5	0.33	0.36
Metier˙FiletMixte˙NordPC	0.1	0.04	0.08	0.27	0.39	0.34	0.36	0.26	0.19	0.21	0.35	0.37
Metier˙FiletMixte˙NordC	0.06	0.0	0.0	0.0	0.01	0.0	0.05	0.0	0.0	0.0	0.01	0.01
Metier˙ChalutMixte˙NordC	0.0	0.0	0.0	0.0	0.0	0.0	0.0	0.0	0.0	0.0	0.0	0.0
Metier˙PalangreMixte˙NordC	0.03	0.0	0.15	0.15	0.15	0.15	0.15	0.15	0.0	0.15	0.15	0.15
Metier˙FiletMerlu˙NordPC	0.07	0.05	0.05	0.02	0.07	0.07	0.08	0.08	0.13	0.09	0.12	0.07

Strategy STR_GdG_Nord_1218m_G

	January	February	March	April	May	June	July	August	September	October	November	December
Metier˙ChalutMixte˙APCS	0.3	0.24	0.2	0.17	0.1	0.1	0.09	0.14	0.18	0.24	0.34	0.35
Metier˙Lang˙NordPC	0.28	0.47	0.47	0.7	0.81	0.79	0.84	0.74	0.6	0.55	0.42	0.41
Metier˙ChalutSole˙NordCet	0.03	0.06	0.02	0.01	0.0	0.0	0.0	0.0	0.0	0.01	0.06	0.02
Metier˙ChalutBenth˙NordAPC	0.39	0.23	0.31	0.12	0.09	0.11	0.07	0.12	0.22	0.2	0.18	0.22
Metier˙FiletSole˙NordC	0.0	0.0	0.0	0.0	0.0	0.0	0.0	0.0	0.0	0.0	0.0	0.0
Metier˙FiletSole˙NordIntPC	0.0	0.0	0.0	0.0	0.0	0.0	0.0	0.0	0.0	0.0	0.0	0.0
Metier˙FiletMixte˙NordPC	0.0	0.0	0.0	0.0	0.0	0.0	0.0	0.0	0.0	0.0	0.0	0.0
Metier˙FiletMixte˙NordC	0.0	0.0	0.0	0.0	0.0	0.0	0.0	0.0	0.0	0.0	0.0	0.0
Metier˙ChalutMixte˙NordC	0.0	0.0	0.0	0.0	0.0	0.0	0.0	0.0	0.0	0.0	0.0	0.0
Metier˙PalangreMixte˙NordC	0.0	0.0	0.0	0.0	0.0	0.0	0.0	0.0	0.0	0.0	0.0	0.0
Metier˙FiletMerlu˙NordPC	0.0	0.0	0.0	0.0	0.0	0.0	0.0	0.0	0.0	0.0	0.0	0.0

Strategy STR_GdG_Nord_1218m_O

	January	February	March	April	May	June	July	August	September	October	November	December
Metier˙ChalutMixte˙NordPC	0.0	0.0	0.0	0.0	0.0	0.0	0.0	0.0	0.0	0.0	0.0	0.0
Metier˙Lang˙NordPC	0.0	0.0	0.0	0.0	0.0	0.0	0.0	0.0	0.0	0.0	0.0	0.0
Metier˙ChalutSole˙NordCet	0.0	0.0	0.01	0.0	0.0	0.0	0.01	0.04	0.01	0.0	0.0	0.0
Metier˙ChalutBenth˙NordC	0.0	0.0	0.0	0.0	0.0	0.0	0.0	0.0	0.0	0.0	0.0	0.0
Metier˙FiletSole˙NordIntPC	0.29	0.59	0.63	0.32	0.43	0.34	0.45	0.43	0.39	0.45	0.22	0.25
Metier˙FiletMixte˙NordC	0.0	0.0	0.0	0.0	0.0	0.0	0.0	0.0	0.0	0.0	0.0	0.0
Metier˙ChalutMixte˙NordC	0.0	0.0	0.01	0.0	0.0	0.0	0.0	0.0	0.01	0.0	0.0	0.0
Metier˙FiletSole˙NordC	0.0	0.0	0.0	0.0	0.0	0.0	0.0	0.0	0.0	0.0	0.0	0.0
Metier˙PalangreMixte˙NordC	0.0	0.0	0.0	0.0	0.0	0.0	0.0	0.0	0.0	0.0	0.0	0.0
Metier˙FiletMerlu˙NordC	0.0	0.0	0.0	0.0	0.0	0.0	0.02	0.0	0.02	0.02	0.0	0.0
Metier˙FiletMixte˙NordPC	0.71	0.41	0.35	0.68	0.57	0.66	0.52	0.53	0.57	0.53	0.78	0.75

Strategy STR_GdG_Nord_moins12m_G

	January	February	March	April	May	June	July	August	September	October	November	December
Metier˙ChalutMixte˙NordPC	0.0	0.0	0.0	0.0	0.0	0.0	0.0	0.0	0.0	0.0	0.0	0.0
Metier˙Lang˙NordPC	0.0	0.0	0.0	0.0	0.0	0.0	0.0	0.0	0.0	0.0	0.0	0.0
Metier˙ChalutSole˙NordCet	0.0	0.0	0.0	0.0	0.0	0.0	0.0	0.0	0.0	0.0	0.0	0.0
Metier˙ChalutBenth˙NordC	0.0	0.0	0.0	0.0	0.0	0.0	0.0	0.0	0.0	0.0	0.0	0.0
Metier˙FiletSole˙NordIntPC	0.0	0.0	0.0	0.0	0.0	0.0	0.0	0.0	0.0	0.0	0.0	0.0
Metier˙FiletMixte˙NordC	0.83	0.72	0.25	0.14	0.0	0.0	0.0	0.0	0.0	0.58	0.54	0.1
Metier˙ChalutMixte˙NordC	0.0	0.0	0.0	0.0	0.0	0.0	0.0	0.0	0.0	0.0	0.0	0.0
Metier˙FiletSole˙NordC	0.09	0.28	0.75	0.0	0.0	0.0	0.0	0.0	0.0	0.35	0.42	0.32
Metier˙PalangreMixte˙NordC	0.08	0.0	0.0	0.86	1.0	1.0	1.0	1.0	1.0	0.07	0.04	0.58
Metier˙FiletMerlu˙NordC	0.0	0.0	0.0	0.0	0.0	0.0	0.0	0.0	0.0	0.0	0.0	0.0
Metier˙FiletMixte˙NordPC	0.0	0.0	0.0	0.0	0.0	0.0	0.0	0.0	0.0	0.0	0.0	0.0

Strategy STR_GdG_Nord_moins12m_G-L

	January	February	March	April	May	June	July	August	September	October	November	December
Metier˙ChalutMixte˙NordPC	0.64	0.29	0.34	0.41	0.47	0.43	0.47	0.55	0.58	0.58	0.57	0.51
Metier˙Lang˙NordPC	0.26	0.53	0.54	0.46	0.46	0.48	0.45	0.37	0.33	0.32	0.27	0.35
Metier˙ChalutSole˙NordCet	0.06	0.18	0.11	0.13	0.07	0.09	0.08	0.07	0.07	0.09	0.14	0.12
Metier˙ChalutBenth˙NordC	0.04	0.0	0.01	0.0	0.0	0.0	0.0	0.01	0.02	0.01	0.02	0.02
Metier˙FiletSole˙NordIntPC	0.0	0.0	0.0	0.0	0.0	0.0	0.0	0.0	0.0	0.0	0.0	0.0
Metier˙FiletMixte˙NordC	0.0	0.0	0.0	0.0	0.0	0.0	0.0	0.0	0.0	0.0	0.0	0.0
Metier˙ChalutMixte˙NordC	0.0	0.0	0.0	0.0	0.0	0.0	0.0	0.0	0.0	0.0	0.0	0.0
Metier˙FiletSole˙NordC	0.0	0.0	0.0	0.0	0.0	0.0	0.0	0.0	0.0	0.0	0.0	0.0
Metier˙PalangreMixte˙NordC	0.0	0.0	0.0	0.0	0.0	0.0	0.0	0.0	0.0	0.0	0.0	0.0
Metier˙FiletMerlu˙NordC	0.0	0.0	0.0	0.0	0.0	0.0	0.0	0.0	0.0	0.0	0.0	0.0
Metier˙FiletMixte˙NordPC	0.0	0.0	0.0	0.0	0.0	0.0	0.0	0.0	0.0	0.0	0.0	0.0

Strategy STR_GdG_Nord_moins12m_O

	January	February	March	April	May	June	July	August	September	October	November	December
Metier˙ChalutBenth˙NordAPC	0.53	0.53	0.67	0.53	0.45	0.56	0.43	0.5	0.66	0.6	0.52	0.55
Metier˙ChalutMixte˙APCS	0.32	0.29	0.21	0.19	0.09	0.08	0.06	0.1	0.14	0.24	0.33	0.31
Metier˙FiletSole˙NordIntPC	0.03	0.03	0.03	0.03	0.03	0.03	0.0	0.0	0.0	0.01	0.03	0.02
Metier˙FiletMixte˙NordInterPC	0.0	0.0	0.0	0.0	0.0	0.01	0.04	0.04	0.04	0.03	0.01	0.01
Metier˙PalangreMixte˙InterC	0.0	0.0	0.0	0.0	0.0	0.0	0.0	0.0	0.0	0.0	0.01	0.01
Metier˙Lang˙NordPC	0.02	0.02	0.05	0.22	0.39	0.29	0.43	0.12	0.12	0.08	0.07	0.08
Metier˙ChalutSole˙NordCet	0.06	0.09	0.0	0.0	0.0	0.0	0.0	0.0	0.0	0.0	0.01	0.0
Metier˙PalangreMerlu˙InterSudAC	0.04	0.04	0.04	0.03	0.04	0.03	0.04	0.04	0.04	0.04	0.02	0.03

Strategy STR_GdG_Nord_plus18m

	January	February	March	April	May	June	July	August	September	October	November	December
Metier˙ChalutMixte˙APCS	0.0	0.0	0.0	0.0	0.0	0.0	0.0	0.0	0.0	0.0	0.0	0.0
Metier˙ChalutSole˙InterSudC	0.0	0.0	0.0	0.0	0.0	0.0	0.0	0.0	0.0	0.0	0.0	0.0
Metier˙Lang˙InterPC	0.0	0.0	0.0	0.0	0.0	0.0	0.0	0.0	0.0	0.0	0.0	0.0
Metier˙FiletSole˙InterSudPC	0.97	0.98	0.91	0.8	0.81	0.83	0.95	0.94	0.93	0.92	0.89	0.91
Metier˙FiletMixte˙InterSudC	0.03	0.02	0.06	0.17	0.16	0.17	0.04	0.05	0.05	0.07	0.07	0.08
Metier˙ChalutBenth˙APCS	0.0	0.0	0.0	0.0	0.0	0.0	0.0	0.0	0.0	0.0	0.0	0.0
Metier˙FiletMerlu˙InterSudPC	0.0	0.0	0.03	0.03	0.02	0.0	0.0	0.01	0.02	0.01	0.02	0.01
Metier˙ChalutBenth˙NordC	0.0	0.0	0.0	0.0	0.0	0.0	0.0	0.0	0.0	0.0	0.0	0.0
Metier˙ChalutSole˙InterC	0.0	0.0	0.0	0.0	0.01	0.0	0.01	0.0	0.0	0.0	0.02	0.0
Metier˙FiletSole˙InterC	0.0	0.0	0.0	0.0	0.0	0.0	0.0	0.0	0.0	0.0	0.0	0.0
Metier˙FiletMIxte˙InterC	0.0	0.0	0.0	0.0	0.0	0.0	0.0	0.0	0.0	0.0	0.0	0.0

Strategy STR_GdG_Sud_1218m_G

	January	February	March	April	May	June	July	August	September	October	November	December
Metier˙ChalutMixte˙APCS	0.92	0.91	0.86	0.59	0.56	0.53	0.55	0.57	0.76	0.86	0.84	0.94
Metier˙ChalutSole˙InterSudC	0.03	0.04	0.02	0.06	0.08	0.04	0.04	0.08	0.09	0.1	0.14	0.02
Metier˙Lang˙InterPC	0.0	0.0	0.0	0.22	0.26	0.35	0.35	0.27	0.11	0.0	0.0	0.0
Metier˙FiletSole˙InterSudPC	0.0	0.0	0.0	0.0	0.0	0.0	0.0	0.0	0.0	0.0	0.0	0.0
Metier˙FiletMixte˙InterSudC	0.0	0.0	0.0	0.0	0.0	0.0	0.0	0.0	0.0	0.0	0.0	0.0
Metier˙ChalutBenth˙APCS	0.05	0.05	0.12	0.13	0.08	0.08	0.06	0.08	0.04	0.04	0.02	0.04
Metier˙FiletMerlu˙InterSudPC	0.0	0.0	0.0	0.0	0.0	0.0	0.0	0.0	0.0	0.0	0.0	0.0
Metier˙ChalutBenth˙NordC	0.0	0.0	0.0	0.0	0.0	0.0	0.0	0.0	0.0	0.0	0.0	0.0
Metier˙ChalutSole˙InterC	0.0	0.0	0.0	0.0	0.0	0.0	0.0	0.0	0.0	0.0	0.0	0.0
Metier˙FiletSole˙InterC	0.0	0.0	0.0	0.0	0.01	0.0	0.0	0.0	0.0	0.0	0.0	0.0
Metier˙FiletMIxte˙InterC	0.0	0.0	0.0	0.0	0.01	0.0	0.0	0.0	0.0	0.0	0.0	0.0

Strategy STR_GdG_Sud_1218m_O

	January	February	March	April	May	June	July	August	September	October	November	December
Metier˙ChaluMixte˙InterC	0.0	0.0	0.0	0.0	0.0	0.0	0.0	0.0	0.0	0.0	0.01	0.0
Metier˙ChalutSole˙InterC	0.0	0.0	0.0	0.0	0.0	0.0	0.0	0.0	0.0	0.0	0.0	0.0
Metier˙FiletMixte˙InterSudC	0.08	0.07	0.12	0.3	0.2	0.09	0.1	0.07	0.1	0.13	0.21	0.15
Metier˙Lang˙InterC	0.0	0.0	0.0	0.0	0.0	0.0	0.0	0.0	0.0	0.0	0.0	0.0
Metier˙FiletSole˙InterSudPC	0.9	0.92	0.86	0.67	0.77	0.89	0.89	0.88	0.86	0.81	0.73	0.78
Metier˙PalangreMixte˙InterC	0.0	0.0	0.0	0.0	0.0	0.0	0.0	0.0	0.0	0.0	0.0	0.0
Metier˙FiletSole˙InterC	0.0	0.0	0.0	0.0	0.0	0.0	0.0	0.0	0.0	0.0	0.0	0.0
Metier˙FiletMIxte˙InterC	0.0	0.0	0.0	0.0	0.0	0.0	0.0	0.0	0.0	0.0	0.0	0.0
Metier˙PalangreMerlu˙InterSudC	0.0	0.0	0.0	0.0	0.0	0.0	0.0	0.0	0.0	0.0	0.0	0.0
Metier˙FiletMerlu˙InterSudC	0.0	0.0	0.0	0.0	0.0	0.0	0.0	0.0	0.0	0.0	0.0	0.0
Metier˙FiletMerlu˙InterSudPC	0.02	0.01	0.02	0.03	0.02	0.01	0.01	0.05	0.04	0.04	0.05	0.06
Metier˙ChalutSole˙InterSudC	0.0	0.0	0.0	0.0	0.01	0.01	0.0	0.0	0.0	0.02	0.0	0.01

Strategy STR_GdG_Sud_moins12m_G

	January	February	March	April	May	June	July	August	September	October	November	December
Metier˙ChaluMixte˙InterC	0.07	0.0	0.0	0.06	0.19	0.38	0.21	0.45	0.22	0.33	0.28	0.28
Metier˙ChalutSole˙InterC	0.0	0.0	0.0	0.02	0.02	0.01	0.08	0.0	0.06	0.06	0.13	0.01
Metier˙FiletMixte˙InterSudC	0.21	0.25	0.15	0.45	0.22	0.07	0.03	0.03	0.15	0.42	0.36	0.17
Metier˙Lang˙InterC	0.0	0.0	0.0	0.0	0.0	0.0	0.0	0.0	0.0	0.0	0.01	0.0
Metier˙FiletSole˙InterSudPC	0.7	0.68	0.74	0.29	0.36	0.3	0.34	0.33	0.16	0.1	0.09	0.49
Metier˙PalangreMixte˙InterC	0.0	0.0	0.0	0.0	0.03	0.04	0.11	0.04	0.14	0.03	0.08	0.0
Metier˙FiletSole˙InterC	0.0	0.0	0.0	0.0	0.0	0.0	0.0	0.0	0.0	0.0	0.0	0.0
Metier˙FiletMIxte˙InterC	0.0	0.0	0.0	0.0	0.0	0.0	0.0	0.0	0.0	0.0	0.0	0.0
Metier˙PalangreMerlu˙InterSudC	0.0	0.0	0.07	0.18	0.17	0.2	0.22	0.13	0.21	0.05	0.0	0.0
Metier˙FiletMerlu˙InterSudC	0.02	0.07	0.04	0.0	0.01	0.0	0.01	0.02	0.06	0.01	0.05	0.05
Metier˙FiletMerlu˙InterSudPC	0.0	0.0	0.0	0.0	0.0	0.0	0.0	0.0	0.0	0.0	0.0	0.0
Metier˙ChalutSole˙InterSudC	0.0	0.0	0.0	0.0	0.0	0.0	0.0	0.0	0.0	0.0	0.0	0.0

Strategy STR_GdG_Sud_moins12m_G-L-O

	January	February	March	April	May	June	July	August	September	October	November	December
Metier˙ChaluMixte˙InterC	0.0	0.0	0.0	0.0	0.0	0.0	0.0	0.0	0.0	0.0	0.0	0.0
Metier˙ChalutSole˙InterC	0.0	0.0	0.0	0.0	0.0	0.0	0.0	0.0	0.0	0.0	0.0	0.0
Metier˙FiletMixte˙InterSudC	0.0	0.0	0.0	0.0	0.0	0.0	0.0	0.0	0.0	0.0	0.0	0.0
Metier˙Lang˙InterC	0.0	0.0	0.0	0.0	0.0	0.0	0.0	0.0	0.0	0.0	0.0	0.0
Metier˙FiletSole˙InterSudPC	0.0	0.0	0.0	0.0	0.0	0.0	0.0	0.0	0.0	0.0	0.0	0.0
Metier˙PalangreMixte˙InterC	0.05	0.0	0.05	0.26	0.62	0.44	0.31	0.32	0.57	0.18	1.0	0.13
Metier˙FiletSole˙InterC	0.0	0.0	0.0	0.0	0.0	0.0	0.0	0.0	0.0	0.0	0.0	0.0
Metier˙FiletMIxte˙InterC	0.0	0.0	0.0	0.0	0.0	0.0	0.0	0.0	0.0	0.0	0.0	0.0
Metier˙PalangreMerlu˙InterSudC	0.95	1.0	0.95	0.74	0.38	0.56	0.69	0.68	0.43	0.82	0.0	0.87
Metier˙FiletMerlu˙InterSudC	0.0	0.0	0.0	0.0	0.0	0.0	0.0	0.0	0.0	0.0	0.0	0.0
Metier˙FiletMerlu˙InterSudPC	0.0	0.0	0.0	0.0	0.0	0.0	0.0	0.0	0.0	0.0	0.0	0.0
Metier˙ChalutSole˙InterSudC	0.0	0.0	0.0	0.0	0.0	0.0	0.0	0.0	0.0	0.0	0.0	0.0

Strategy STR_GdG_Sud_moins12m_L

	January	February	March	April	May	June	July	August	September	October	November	December
Metier˙ChaluMixte˙InterC	0.93	0.95	0.88	0.74	0.79	0.76	0.75	0.8	0.88	0.85	0.81	0.81
Metier˙ChalutSole˙InterC	0.07	0.05	0.11	0.24	0.19	0.24	0.25	0.2	0.12	0.14	0.19	0.16
Metier˙FiletMixte˙InterSudC	0.0	0.0	0.0	0.0	0.0	0.0	0.0	0.0	0.0	0.0	0.0	0.0
Metier˙Lang˙InterC	0.0	0.0	0.0	0.02	0.02	0.0	0.0	0.0	0.0	0.01	0.0	0.03
Metier˙FiletSole˙InterSudPC	0.0	0.0	0.0	0.0	0.0	0.0	0.0	0.0	0.0	0.0	0.0	0.0
Metier˙PalangreMixte˙InterC	0.0	0.0	0.0	0.0	0.0	0.0	0.0	0.0	0.0	0.0	0.0	0.0
Metier˙FiletSole˙InterC	0.0	0.0	0.01	0.0	0.0	0.0	0.0	0.0	0.0	0.0	0.0	0.0
Metier˙FiletMIxte˙InterC	0.0	0.0	0.0	0.0	0.0	0.0	0.0	0.0	0.0	0.0	0.0	0.0
Metier˙PalangreMerlu˙InterSudC	0.0	0.0	0.0	0.0	0.0	0.0	0.0	0.0	0.0	0.0	0.0	0.0
Metier˙FiletMerlu˙InterSudC	0.0	0.0	0.0	0.0	0.0	0.0	0.0	0.0	0.0	0.0	0.0	0.0
Metier˙FiletMerlu˙InterSudPC	0.0	0.0	0.0	0.0	0.0	0.0	0.0	0.0	0.0	0.0	0.0	0.0
Metier˙ChalutSole˙InterSudC	0.0	0.0	0.0	0.0	0.0	0.0	0.0	0.0	0.0	0.0	0.0	0.0

Strategy STR_GdG_Sud_moins12m_O

	January	February	March	April	May	June	July	August	September	October	November	December
Metier˙ChalutMixte˙APCS	0.0	0.0	0.0	0.01	0.04	0.0	0.0	0.0	0.0	0.0	0.0	0.0
Metier˙FiletMerlu˙InterSudAPC	0.48	0.49	0.68	0.48	0.49	0.45	0.52	0.34	0.4	0.41	0.34	0.36
Metier˙FiletMixte˙NordInterPC	0.01	0.01	0.05	0.17	0.13	0.19	0.09	0.07	0.05	0.11	0.19	0.09
Metier˙ChalutSole˙InterSudC	0.0	0.0	0.0	0.0	0.0	0.0	0.0	0.0	0.0	0.0	0.0	0.0
Metier˙ChalutBenth˙APCS	0.0	0.0	0.0	0.0	0.0	0.0	0.0	0.0	0.0	0.0	0.0	0.0
Metier˙FiletSole˙InterSudPC	0.51	0.5	0.27	0.34	0.31	0.36	0.39	0.59	0.55	0.47	0.46	0.55
Metier˙Lang˙InterPC	0.0	0.0	0.0	0.0	0.0	0.0	0.0	0.0	0.0	0.0	0.0	0.0
Metier˙FiletMIxte˙InterC	0.0	0.0	0.0	0.0	0.0	0.0	0.0	0.0	0.0	0.0	0.0	0.0
Metier˙ChalutSole˙InterC	0.0	0.0	0.0	0.0	0.03	0.0	0.0	0.0	0.0	0.01	0.01	0.0

Strategy STR_GdG_Sud_plus18m_G

	January	February	March	April	May	June	July	August	September	October	November	December
Metier˙ChalutMixte˙APCS	0.93	0.9	0.49	0.43	0.33	0.26	0.43	0.54	0.68	0.74	0.87	0.87
Metier˙FiletMerlu˙InterSudAPC	0.0	0.0	0.0	0.0	0.0	0.0	0.0	0.0	0.0	0.0	0.0	0.0
Metier˙FiletMixte˙NordInterPC	0.0	0.0	0.0	0.0	0.0	0.0	0.0	0.0	0.0	0.0	0.0	0.0
Metier˙ChalutSole˙InterSudC	0.01	0.01	0.0	0.01	0.02	0.03	0.04	0.05	0.11	0.09	0.04	0.02
Metier˙ChalutBenth˙APCS	0.06	0.09	0.51	0.53	0.59	0.53	0.32	0.27	0.18	0.17	0.09	0.11
Metier˙FiletSole˙InterSudPC	0.0	0.0	0.0	0.0	0.0	0.0	0.0	0.0	0.0	0.0	0.0	0.0
Metier˙Lang˙InterPC	0.0	0.0	0.0	0.03	0.06	0.18	0.21	0.14	0.03	0.0	0.0	0.0
Metier˙FiletMIxte˙InterC	0.0	0.0	0.0	0.0	0.0	0.0	0.0	0.0	0.0	0.0	0.0	0.0
Metier˙ChalutSole˙InterC	0.0	0.0	0.0	0.0	0.0	0.0	0.0	0.0	0.0	0.0	0.0	0.0

Strategy STR_GdG_Sud_plus18m_O

Table S21: Proportion of each strategy (table) exerced by a *métier* (row) during a month (column)

Temporal effort variations

year	season	GBOB	LBOB	TFBE	TFBW	TNEP
2010	1	1	1	1	1	1
2010	2	1	1	1	1	1
2010	3	1	1	1	1	1
2010	4	1	1	1	1	1
2011	1	1.25418000711491	1.50802271044187	0.822485207100592	0.736613847743713	1.23900622397682
2011	2	1.35299869621904	1.53417431192661	1.14337568058076	0.545869804919423	1.01593013668345
2011	3	1.84072366087265	1.0467180174146	1	0.61088318085855	0.861817841365521
2011	4	1.24739676840215	1.13833812622976	0.753443526170799	0.79576967648479	0.458688962655027
2012	1	1.12611170401992	1.9607504319921	1.04497041420118	0.354798484326559	0.762853352355315
2012	2	1.73696219035202	1.45412844036697	1.23865698729583	0.48430449533503	0.816151615221419
2012	3	1.41149343738915	0.932853315472204	1.17028670721112	0.820119634060521	0.663422144726397
2012	4	1.63985637342908	1.12850007567731	0.636363636363636	0.404109126026074	0.56100547834478
2013	1	1.00480256136606	1.24611207109356	1.16686390532544	0.625504960385808	3.57668932033879
2013	2	1.42405475880052	0.972018348623853	2.15154264972777	0.436670623409669	1.6245388562652
2013	3	1.35083362894643	0.842096450100469	1.47871416159861	0.703777178043631	0.942133066548336
2013	4	1.41687612208259	1.05766611169971	1.02203856749311	0.439101670690488	0.865424559393202
2014	1	1.08929206688011	1.28634904961738	3.17396449704142	0.213117464691698	2.68168431751125
2014	2	1.69067796610169	1.36651376146789	2.90199637023593	0.313210347752332	0.953570002494243
2014	3	1.99255054984037	1.15003348961822	1.49782797567333	0.389071076706545	0.788242402724236
2014	4	2.01795332136445	1.24897835628878	1.45316804407714	0.479879285369387	0.793013926520409
2015	1	0.405016008537887	2.74401382374722	4.40591715976331	0.497361350327248	1.61165630832504
2015	2	1.83050847457627	1.60756880733945	3.28312159709619	0.435818490245971	1.50487722496828
2015	3	1.77899964526428	1.34611520428667	1.38053866203301	0.621871921182266	0.811258285704819
2015	4	1.42477558348294	1.62191614953837	1.7396694214876	0.790656687590536	0.930883335383297
2016	1	1.21095695482035	1.63317699333498	3.52189349112426	0.420936961763693	3.03581452560523
2016	2	1.94980443285528	1.65733944954128	4.31669691470054	0.460555555555556	1.38022556962673
2016	3	2.42213550904576	1.35884125920965	1.68549087749783	0.562047853624208	1.91003274640718
2016	4	1.93824057450628	1.4277281670955	2.26997245179063	0.577754707870594	0.776020232421546
2017	1	0.90175501007945	1.88784662223319	3.70059171597633	0.37713859226088	2.44305171714717
2017	2	2.07936766623211	1.80275229357802	4.67037205081682	0.403194797851286	1.27955759902975
2017	3	2.63692798864857	1.46324514400538	1.52128583840139	0.524330283837673	1.16984447827875
2017	4	1.79365649311789	1.43287422430755	2.47417355371908	0.616096893610172	0.833305831441751
2018	1	0.90175501007945	1.88784662223319	3.70059171597633	0.37713859226088	2.44305171714717
2018	2	2.20893089960894	1.94816513761475	5.0240471869331	0.403194797851286	1.27955759902975
2018	3	2.85172046825138	1.56764902880112	1.52128583840139	0.524330283837673	1.16984447827875
2018	4	1.79365649311789	1.43287422430755	2.67837465564752	0.616096893610172	0.833305831441751
2019	1	0.90175501007945	1.88784662223319	3.70059171597633	0.37713859226088	2.44305171714717
2019	2	2.33849413298578	2.09357798165148	5.37772232304938	0.403194797851286	1.27955759902975
2019	3	3.06651294785418	1.67205291359685	1.52128583840139	0.524330283837673	1.16984447827875
2019	4	1.79365649311789	1.43287422430755	2.88257575757597	0.616096893610172	0.833305831441751
2020	1	0.90175501007945	1.88784662223319	3.70059171597633	0.37713859226088	2.44305171714717
2020	2	2.46805736636261	2.23899082568821	5.73139745916566	0.403194797851286	1.27955759902975
2020	3	3.28130542745699	1.77645679839259	1.52128583840139	0.524330283837673	1.16984447827875
2020	4	1.79365649311789	1.43287422430755	3.08677685950441	0.616096893610172	0.833305831441751

Table S22: Standardized effort coefficients of variation $VarsEff_{met,s,y}$, by *métier* group, year and season in ISIS-Fish for hake (GBOB: netters ; LBOB: longliners ; TFBE: whitefish French trawlers ; TFBW: Spanish trawlers ; TNEP: Norway lobster trawlers)

year	GBOB	TFBE
2010	1	1
2011	1.37926067745381	1.06255328883066
2012	1.22968103918206	0.990815258455161
2013	1.35373760741716	0.99641621782741
2014	1.183970856102	0.997150997150997
2015	1.17111846799534	0.785035456568303
2016	1.0083717797012	0.742210299387915
2017	0.964472010600987	0.678475124947129
2018	0.920572241500769	0.614739950506343
2019	0.876672472400552	0.551004776065557
2020	0.832772703300334	0.487269601624771

Table S23: Standardized effort coefficients of variation $VarsEff_{met,s,y}$, by *métier* group, year and season in ISIS-Fish for sole (GBOB: netters ; TFBE: whitefish French trawlers)

year	TNEP
2010	1
2011	1.03188529852343
2012	0.756045367001926
2013	0.834581639203937
2014	0.88444254226407
2015	1.15364861973037
2016	1.41707682430987
2017	1.55023539482136
2018	1.68339396533285
2019	1.81655253584434
2020	1.94971110635583

Table S24: Standardized effort coefficients of variation $VarsEff_{met,s,y}$, by *métier* group, year and season in ISIS-Fish for Norway lobster (TNEP: Norway lobster trawlers)

Target factors

Metier	estimate
PTBV˙VIIIabd	0.76
OBTS˙VIIIabd	0.13
Metier˙ChalutMixte˙NordPC	1.52
Metier˙ChaluMixte˙InterC	1.11
Metier˙ChalutBenth˙NordAPC	0.36
Metier˙ChalutBenth˙NordC	2.07
Metier˙ChalutBenth˙APCS	0.72
Metier˙Lang˙NordPC	1.62
Metier˙Lang˙InterC	3.4
Metier˙FiletSole˙NordC	0.01
Metier˙FiletSole˙NordIntPC	0.02
Metier˙FiletSole˙InterSudPC	0.02
Metier˙FiletSole˙InterC	0.08
Metier˙ChalutSole˙InterC	1.52
Metier˙PalangreMerlu˙InterSudAC	12.14
Metier˙PalangreMerlu˙InterSudC	7.13
Metier˙PalangreMixte˙NordC	0.26
Metier˙PalangreMixte˙InterC	0.41
Metier˙ChalutSole˙InterSudC	1.46
Metier˙ChalutSole˙NordCet	0.54
Metier˙FiletMerlu˙NordC	0.38
Metier˙FiletMerlu˙NordPC	1.49
Metier˙FiletMixte˙NordPC	1.0
Metier˙FiletMixte˙NordC	1.0
Metier˙FiletMixte˙NordInterPC	1.0
Metier˙FiletMixte˙InterSudC	0.13
Metier˙FiletMIxte˙InterC	0.13
Metier˙FiletMerlu˙InterSudAPC	1.64
Metier˙FiletMerlu˙InterSudC	0.18
Metier˙ChalutMixte˙NordC	0.66
Metier˙FiletMerlu˙InterSudPC	0.28
Metier˙ChalutMixte˙APCS	1.05
Metier˙Lang˙InterPC	2.84

Table S25: *Métier* component of the fixed component of target factors for hake

Metier	s1	s2	s3	s4
Metier˙ChalutMixte˙NordPC	0.20302651	0.14858184	0.39940007	0.24899158
Metier˙ChaluMixte˙InterC	0.17374381	0.24780502	0.35249127	0.22595990
Metier˙ChalutBenth˙NordAPC	0.30268436	0.19839603	0.25242189	0.24649772
Metier˙ChalutBenth˙NordC	0.35127559	0	0.24801211	0.40071230
Metier˙ChalutBenth˙APCS	0.30939923	0.20472724	0.20517176	0.28070178
Metier˙ChalutSole˙InterC	0.21313671	0.15845812	0.48381653	0.14458864
Metier˙ChalutSole˙InterSudC	0.19457849	0.18870437	0.35707480	0.25964234
Metier˙ChalutSole˙NordCet	0.20500053	0.22211697	0.32998304	0.24289945
Metier˙ChalutMixte˙NordC	0	0.63240873	0.30365804	0.06393323
Metier˙ChalutMixte˙APCS	0.21890302	0.29368301	0.32154643	0.16586753
Metier˙Lang˙InterC	0	0.33220706	0.47340530	0.19438764
Metier˙Lang˙NordPC	0.11481646	0.31815005	0.42072550	0.14630799
Metier˙Lang˙InterPC	0.09210562	0.26977737	0.36112641	0.27699059
OBTS˙VIIIabd	0.37981386	0.25277468	0.15096154	0.21644992
PTBV˙VIIIabd	0.37981386	0.25277468	0.15096154	0.21644992
Metier˙FiletSole˙NordC	0.20241465	0	0	0.79758535
Metier˙FiletSole˙NordIntPC	0.23571628	0.21186004	0.22131441	0.33110927
Metier˙FiletSole˙InterSudPC	0.28907103	0.18612597	0.25444936	0.27035364
Metier˙FiletSole˙InterC	0	1	0	0
Metier˙FiletMerlu˙NordC	0.10760827	0.41139521	0.19920891	0.28178760
Metier˙FiletMerlu˙NordPC	0.25929910	0.23170453	0.28261319	0.22638318
Metier˙FiletMixte˙NordPC	0.16464438	0.22575187	0.32319540	0.28640836
Metier˙FiletMixte˙NordC	0.09459163	0.21704363	0.56107359	0.12729115
Metier˙FiletMixte˙NordInterPC	0.07365546	0.06250456	0.03759845	0.82624153
Metier˙FiletMixte˙InterSudC	0.23095464	0.17185774	0.22880568	0.36838194
Metier˙FiletMIxte˙InterC	0	0.30095321	0.02149133	0.67755547
Metier˙FiletMerlu˙InterSudAPC	0.36550647	0.15792438	0.23455184	0.24201731
Metier˙FiletMerlu˙InterSudC	0.30629689	0.40188062	0.25083899	0.04098351
Metier˙FiletMerlu˙InterSudPC	0.18991872	0.16774025	0.16016415	0.48217689
Metier˙PalangreMerlu˙InterSudAC	0.18289224	0.32730446	0.25396035	0.23584295
Metier˙PalangreMixte˙NordC	0.18637272	0.47541596	0.19785392	0.14035740
Metier˙PalangreMixte˙InterC	0.59384064	0.11543074	0.23814271	0.05258590
Metier˙PalangreMerlu˙InterSudC	0.21953798	0.37435633	0.25660379	0.14950190

Table S26: Season component of the fixed component of target factors for hake

Species : *Métier* ISIS-Fish	Fixed component of the target factor
Sole : Metier_FiletSole_NordC	0.66
Sole : Metier_FiletSole_NordIntPC	0.9295566
Sole : Metier_FiletSole_InterSudPC	1.04
Sole : Metier_FiletSole_InterC	0.30
Sole : Metier_FiletMerlu_NordPC	0.02
Sole : Metier_FiletMixte_NordPC	0.14
Sole : Metier_FiletMixte_NordC	0.18
Sole : Metier_FiletMixte_NordInterPC	0.35
Sole : Metier_FiletMixte_InterSudC	0.18
Sole : Metier_FiletMIxte_InterC	0.18
Sole : Metier_FiletMerlu_InterSudAPC	0.01
Sole : Metier_FiletMerlu_InterSudC	0.03
Sole : Metier_FiletMerlu_InterSudPC	0.22
Sole : Metier_ChalutMixte_NordPC	3.93
Sole : Metier_ChaluMixte_InterC	5.42
Sole : Metier_ChalutBenth_NordAPC	0.58
Sole : Metier_ChalutBenth_NordC	2.62
Sole : Metier_ChalutBenth_APCS	0.1
Sole : Metier_ChalutSole_InterC	20.70
Sole : Metier_ChalutSole_InterSudC	23.92
Sole : Metier_ChalutMixte_NordC	8.24
Sole : Metier_ChalutMixte_APCS	4.05
Sole : Metier_Lang_InterC	12.08
Sole : Metier_Lang_NordPC	2.74
Sole : Metier_Lang_InterPC	4.31
Norway lobster : Metier_ChalutMixte_NordPC	8.25
Norway lobster : Metier_ChaluMixte_InterC	4.15
Norway lobster : Metier_ChalutBenth_NordAPC	0.92
Norway lobster : Metier_ChalutBenth_NordC	4.96
Norway lobster : Metier_ChalutBenth_APCS	0.21
Norway lobster : Metier_ChalutSole_InterC	3.34
Norway lobster : Metier_ChalutSole_InterSudC	2.56
Norway lobster : Metier_ChalutMixte_NordC	2.76
Norway lobster : Metier_ChalutMixte_APCS	4.85
Norway lobster : Metier_Lang_InterC	26.56
Norway lobster : Metier_Lang_NordPC	39.09
Norway lobster : Metier_Lang_InterPC	26.80

Table S27: Fixed component of target factors for sole and Norway lobster

Métiers group	Quarter 1	Quarter 2	Quarter 3	Quarter 4
Longliners	2.4220384	1.5861784	2.5610928	1.822776
Gillnetters	105.229808	62.207856	57.04024	33.275344
French whitefish trawlers	0.77101264	1.02484632	0.74847584	1.04700328
Spanish whitefish trawlers	6.279988	11.3637192	12.8470736	11.6260096
Norway lobster trawlers	7.846248	12.340336	14.063496	43.476832

Table S28: Estimated component of target factors for hake, for each quarter (column) and *métiers* group (rows)

Métiers group	Quarter 1	Quarter 2	Quarter 3	Quarter 4
Gillnetters	15.82761	7.970654	8.516764	13.45805
French whitefish trawlers	0.1606412	0.1556396	0.2472493	0.2695311
Norway lobster trawlers	1.054958	0.5972864	0.5024087	0.986959

Table S29: Estimated component of target factors for sole, for each quarter (column) and *métiers* group (rows)

French whitefish trawlers	Norway lobster trawlers
0.06314853	1.175236

Table S30: Estimated component of target factors for Norway lobster, for each group of *métiers* (column)

A.3 Management

Reducing effort and discards when the TAC is nearly reached Condition to consider if TAC is near-reached during year y at the beginning of month m (numbering beginning at 1): the expected catch this month (the monthly mean over the current year) is bigger than the remaining catch.

$$\frac{\sum_{t=1}^{m-1} C_{sp,t}}{m-1} > TAC_{sp,y} - \sum_{t=1}^{m-1} C_{sp,t} \tag{20}$$

The above condition is verified each time-step from 2013, before *métier* flagging, until the TAC is considered near reached or reached. The catch is either the landings or the catch, depending on whether the TAC is a TAL or a TAC (always a TAL for *nephrops*; TAL for hake and sole until 2015, TAC then). If the TAC/TAL is considered near reached at step m, it is automatically considered reached at step $m+1$, whether it is actually reached.

Ratio to apply to the month-strategy-*métier* proportion:

$$r_{prop} = \frac{TAC_{sp,y} - \sum_{t=1}^{m-1} C_{sp,t}}{\frac{\sum_{t=1}^{m-1} C_{sp,t}}{m-1}} \tag{21}$$

One ratio is derived per species whose TAC is considered near reached. For each *métier* catching several of these species, the samllest ratio is to be applied to the corresponding strategy-*métier*-month proportion.

Once the TAC is considered reached, only if the landing obligation applies, a few more calculations are done in order to limit the discards during the first month of reached TAC (reducing an artifact). these are done just after the re-allocation of forbidden *métiers* effort.

Computing the allowed discards this month:

$$D^{allowed}_{sp,m} = exemption_{sp,met} \sum_{t=1}^{m-1} C_{sp,t} - \sum_{t=1}^{m-1} D_{sp,t} \tag{22}$$

Computing the ratio to be applied to an effort value:

$$r_{disc,sp} = \frac{D^{allowed}_{sp,m}}{\frac{\sum_{t=1}^{m-1} C_{sp,t}}{m_T}} \tag{23}$$

with m_T the last month when no TAC was reached or near reached. Here m_T and $D^{allowed}_{sp,m}$ are not derived on the same duration, because almost no catch happens after the TAC is reached and/or near reached, and also beacause using m instead of m_T increases r_{disc}, and hence worsens the discarding artifact.

This ratio is computed for each species. *Métiers* catching several species use the smallest ratio.

A maximal strategy-*métier*-month proportion is derived as following:

$$StrMetMonth^{max}_{str,met,m} = r_{disc,sp} \sum_{t=1}^{m_T} StrMetMonth_{str,met,t} \tag{24}$$

The strategy-*métier*-month proportions are then capped:

$$StrMetMonth_{str,met,m} = \begin{cases} StrMetMonth^{max}_{str,met,m}, \ StrMetMonth_{str,met,m} \geq StrMetMonth^{max}_{str,met,m} \\ StrMetMonth_{str,met,m}, \ StrMetMonth_{str,met,m} < StrMetMonth^{max}_{str,met,m} \end{cases} \tag{25}$$

This capping occurs after effort has potentially been re-allocated to *métier met*.

Ideally, this capping should ensure that the *de minimis* exemptions are respected, but given q, $Tarf$ and biomass variations, more discards occur. It is however still less than before these calculations. Also, some *métiers* may have no effort allocated to them at the beginning of year; but not at the end. Hence, they are forbidden, and maybe re-allocated effort late in the year, explaining why marginal discarding may occur months after the TAC was reached.

Adapting effort to the TAC value Since the previous correction is not enough to eliminate all the artifacts, the following has been implemented. The idea is that the effort is altered for the whole year once the TAC has been derived to prevent extreme overshooting or undershooting (done once per year, in January, from 2013). Here catch and TAC are used irregardless of working on landings and TALs (hake and sole before 2016, *Nephrops*) or catch and TACs (hake and sole from 2016).

The idea is to compute for each year for CPUE in the Bay of Biscay for each stock:

$$CPUE_{BoB,s,y} = \frac{C_{BoB,s,y}}{Ey} \tag{26}$$

Effort is only accounted for *métiers* catching the stock s. The catch of Spanish longliners and gillnetters is accounted for hake, but not their effort. Hence, for hake, it is a catch per unit of effort not coming from the Spanish longliners and gillnetters; still, this is enough to correct the effort.

For each stock, a mean CPUE over years is then derived:

$$CPU\bar{E}_{BoB,s} = \frac{\sum_{y} CPUE_{BoB,s,y}}{num\ years} \tag{27}$$

From which an expected catch is derived, using what we already know on expected effort for the current year (equals 2010 effort, times the inter-annual variations of effort for the current year, at the gear level):

$$C_{s,y}^{exp} = CPU\bar{E}_{BoB,s} * E(y) \tag{28}$$

Then a ratio is derived:

$$r_s = \frac{TAC}{C_{s,y}^{exp}} \tag{29}$$

And the inter-annual variation components of the target factors are accordingly altered:

$$VarsEff_{met,s,y}^{NEW} = r_s * VarsEff_{met,s,y}^{OLD} \tag{30}$$

For each *métier* met, the ratio r_s is:

- The smallest of all ratios if at least one stock is expected to be overshot (at least one ratio is smaller than 1)
- The biggest of all ratios if none stock is expected to be overshot (all ratios bigger or equal than/to 1)

References

Alvarez, P., Fives, J., Motos, L., and Santos, M. (2004). Distribution and abundance of European hake Merluccius merluccius (L.), eggs and larvae in the North East Atlantic waters in 1995 and 1998 in relation to hydrographic conditions. *Journal of Plankton Research*, 26(7):811–826.

Casey, J. and Pereiro, J. (1995). European hake (M. merluccius) in the North-east Atlantic. In *Hake*, pages 125–147. Springer.

Conan, G. (1975). Périodicité des mues, croissance et cycle biologique de nephrops norvegicus dans le golfe de gascogne. *Comptes rendus de l'Académie des sciences de Paris*, 281(18):1349–1352.

Conan, G. (1978). Average growth curves and life history in a Nephrops norvegicus population from Northern Bay of Biscay. *ICES CM.*

Deporte, N., Ulrich, C., Mahevas, S., Demaneche, S., and Bastardie, F. (2012). Regional metier definition: a comparative investigation of statistical methods using a workflow applied to international otter trawl fisheries in the North Sea. *ICES Journal of Marine Science*, 69(2):331–342.

Drouineau, H. (2008). *Développement et ajustement d'un modèle de dynamique des populations structuré en longueur et spatialisé appliqué au stock Nord de merlu (Merluccius merluccius).* PhD thesis, École Nationale Supérieure d'Agronomie de Rennes.

Fabens, A. J. (1965). Properties and fitting of the von bertalanffy growth curve. *Growth*, 29:265–289.

Guichet, R. (1996). Le merlu Européen (Merluccius merluccius L.). Rapport interne DRV. IFREMER RH / L'Houmeau.

ICES (1991). Report of the Working Group on the Assessment of the Stocks of Hake. ICES CM 1991/Assess: 20. 181 pp.

ICES (2006). Report of the Benchmark Workshop on Nephrops Stocks(WKNEPH), 24-27 January 2006, ICES HQ, Copenhagen, Denmark. ICES CM 2006/ACFM:12. 85 pp.

ICES (2010). Report of the Benchmark Workshop on Roundfish (WKROUND), 9-16 February 2010, Copenhagen, Denmark. ICES CM 2010/ACOM:36. 183 pp.

ICES (2013). Report of the Working Group on the Assessment of Southern Shelf Stocks of Hake, Monk and Megrim (WGHMM),10-16 May 2013, ICES HQ, Copenhagen, Denmark. ICES CM 2013/ACOM:11A. 7227 pp.

ICES (2014a). Report of the Benchmark Workshop on Southern megrim and hake (WKSOUTH), 3-7 February 2014, ICES HQ, Copenhagen, Denmark. ICES CM 2014/ACOM:40. 236 pp.

ICES (2014b). Report of the Working Group for the Bay of biscay and the Iberian waters Ecoregion (WGBIE), 7-13 may 2014, lisbon, portugal. ICES CM 2014/ACOM:11. 714 pp.

ICES (2016). Report of the Benchmark Workshop on Nephrops Stocks(WKNEP), 24-28 October 2016, Cadiz, Spain. ICES CM 2016/ACOM:38. 223 pp.

ICES (2017). Report of the Working Group for the Bay of Biscay and the Iberian waters Ecoregion (WGBIE). 4-19 May 2017, ICES HQ, Cadiz, Spain. ICES CM/ACOM:12 532 pp.

Marchal, P. (2005). Technological developments and tactical adaptations of important EU fleets (TECTAC).

Methot, Jr., R. D. and Wetzel, C. R. (2013). Stock synthesis: A biological and statistical framework for fish stock assessment and fishery management. *Fisheries Research*, 142(SI):86–99.

Morizur, Y. (1982). Estimation de la mortalité pour quelques stocks de langoustine, Nephrops norvegicus. In *Council Meeting, 1982, of the International Council for the Exploration of the Sea,(Copenhagen (Denmark)).*

Pardo, S., Hess, P., Simon, E., Barille, L., Geslin, E., Cognie, B., Martin-Jezequel, V., Sechet, V., Herrenknecht, C., Baron, R., et al. (2017). Coselmar. compréhension des socio-écosystèmes littoraux et marins pour la prévention et la gestion des risques. bilan scientifique 2013-2017. http://archimer.ifremer.fr/doc/00406/51793/ , consulté le 07/05/2018.

Pelletier, D., Mahévas, S., Drouineau, H., Vermard, Y., Thebaud, O., Guyader, O., and Poussin, B. (2009). Evaluation of the bioeconomic sustainability of multi-species multi-fleet fisheries under a wide range of policy options using ISIS-Fish. *Ecological Modelling*, 220:1013–1033.

Poulard, J. (2001). Distribution of hake (Merluccius merluccius, Linnaeus, 1758) in the Bay of Biscay and the Celtic sea from the analysis of French commercial data. *Fisheries Research*, 50(1-2):173–187.

Sala, A., Bastardie, F., De Carlo, F., Dinesen, G., Eigaard, O., Feekings, J., Frandsen, R., Jonsson, P., Krag, L., Laffargue, P., Magnusson, M., Nielsen, J., Notti, E., Papadopoulo, N., Polet, H., Rijnsdorp, A., Sköld, M., Smith, C., van Marlen, B., Virgilli, M., and Zengin, M. (2014). Report on options for mitigation fishing impacts in regional seas.

Seber, G. A. F. (1982). The estimation of animal abundance.

Vigier, A. (2018). *Développement d'une plateforme d'évaluation de stratégies de gestion spatialisés : application au stock de merlu Nord d'Atlantique Nord-Est et à la pêcherie mixte démersale du golfe de Gascogne*. PhD thesis, Université de Bretagne Occidentale.

Vigier, A., Mahévas, S., and Bertignac, M. (2018). Towards a spatial integrated stock assessment model for european hake northern stock. *Fisheries Research*, 199:158–170.

von Bertalanffy, L. (1938). A quantitative theory of organic growth (inquiries on growth laws. ii). *Human biology*, 10(2):181–213.

Woillez, M., Poulard, J.-C., Rivoirard, J., Petitgas, P., and Bez, N. (2007). Indices for capturing spatial patterns and their evolution in time, with application to European hake (Merluccius merluccius) in the Bay of Biscay. *ICES Journal of Marine Science*, 64(3):537–550.

Worsøe Clausen, L. et al. (2016). Myfish d2.4 – scientific advances on msy and the implications for management (month 48).
